# MNX1-AS1, a c-Myc induced lncRNA, promotes the Warburg effect by regulating PKM2 nuclear translocation

**DOI:** 10.1186/s13046-022-02547-3

**Published:** 2022-12-07

**Authors:** Yang Wu, Yichun Wang, Hanhui Yao, Heng Li, Fanzheng Meng, Qidong Li, Xiansheng Lin, Lianxin Liu

**Affiliations:** 1 Cheeloo College of Medicine, Shandong University, Jinan, 250002 China; 2 Department of Hepatobiliary Surgery, The First Affiliated Hospital of USTC, Division of Life Sciences and Medicine, University of Science and Technology of China, Anhui Province Key Laboratory of Hepatopancreatobiliary Surgery, Anhui Provincial Clinical Research Center for Hepatobiliary Diseases, Hefei, 230001 China

**Keywords:** MNX1-AS1, c-Myc, PKM2, EGF, Warburg effect

## Abstract

**Background:**

Altered glycolysis is the most fundamental metabolic change associated with the Warburg effect. Some glycolytic enzymes such as PKM2, the dominant pyruvate kinase in cancer cells, have been shown to engage in non-glycolytic functions that contribute to tumor metabolism. However, the precise mechanisms are not completely understood.

**Methods:**

The role of MNX1-AS1 in hepatocellular carcinoma progression was assessed both in vitro and in vivo. Northern blotting, RNA pulldown, mass spectrometry, RNA-binding protein immunoprecipitation, ChIP, luciferase reporter assays, RNA FISH and immunofluorescence staining were used to explore the detail molecular mechanism of MNX1-AS1 in hepatocellular carcinoma (HCC).

**Results:**

Here we dissect how MNX1-AS1, a long non-coding RNA (lncRNA), reinforces the Warburg effect through facilitating the non-glycolytic actions of PKM2 in the cell nucleus. We found that MNX1-AS1 expression was frequently overexpressed in HCC-derived cell lines and tissues compared to their normal hepatic cell counterparts, a finding consistent with its status as pan-cancer expressed lncRNA. In the context of HCC, we show MNX1-AS1 acts as a scaffold to promote interactions between PKM2 and importin α5. In response to EGFR activation, the resulting ternary complex drives the translocation of PKM2 into the nucleus. In consequence, glycolytic pathway components including key mediators of the Warburg effect (LDHA, GLUT1 and PDK1) are upregulated though the coactivator function of PKM2. Manipulating MNX1-AS1 elicited robust effects on glycolysis associated with marked changes in HCC growth in vitro and in xenograft models, indicative of the significant contribution of MNX1-AS1 to tumorigenic phenotypes. Moreover, while MNX1-AS1 expression is driven by c-Myc, its actions associated with PKM2 were shown to be downstream and independent of c-Myc.

**Conclusions:**

Given the status of MNX1-AS1 as a pan-cancer upregulated lncRNA, this implicitly highlights the potential of targeting MNX1-AS1 to selectively counter the Warburg effect in a range of tumor types.

**Supplementary Information:**

The online version contains supplementary material available at 10.1186/s13046-022-02547-3.

## Background

Several metabolic enzymes have been found to act outside of their classical roles. For example, the glycolytic enzyme pyruvate kinase isoform M2 (PKM2) has been shown to engage in non-metabolic activities including coactivator and protein kinase functions [1]. Notably, these alternate roles can contribute to tumor progression with another glycolytic enzyme, 6-phosphofructo-2-kinase/fructose-2,6-bisphosphatase 4 (PFKFB4) shown to phosphorylate SRC-3 resulting in its transcriptional activation which was shown to drive breast tumor growth and metastasis [2]. Moreover, 3-phosphoglycerate dehydrogenase (PHGDH), which interconverts 3-phosphoglycerate (3-PG) to 3-phosphohydroxypyruvate (3-PHP) during serine biosynthesis, was recently reported to sustain tumor growth upon glucose restriction [3]. Nevertheless, the detailed mechanisms by which metabolic enzymes support tumor progression through their non-metabolic functions are still poorly understood.

Pyruvate kinases are responsible for the final rate-limiting step of glycolysis, catalyzing the conversion of phosphoenolpyruvate (PEP) to pyruvate to yield one molecule of ATP [4]. Mammalian cells possess two distinct pyruvate kinase genes which generate four isozymes [5]. PKR and PKL transcripts are derived from different promoters in the *PKL* gene whereas PKM1 and PKM2 result from alternative splicing of *PKM.* PKM2 is the dominant isoform during mammalian embryogenesis but its expression is replaced during development by tissue-specific isozymes, namely PKM1, PKL and PKR. Intriguingly, cancer cells often exhibit re-expression of PKM2 with diminished expression of PKM1/L/R [6, 7]. Indeed, PKM2 is broadly upregulated in epithelial cancers derived from the liver, colon, lung, kidney, breast, papillary thyroid, cervix, prostate and bladder [8–10]. Glycolysis is a cytoplasmic process and PKM2 is predominantly localized to the cytoplasm. However, a pool of PKM2 also exists in the nucleus which appears to make substantial contributions to tumorigenesis. For instance, nucleus PKM2 interacts with HIF1α to enhance the expression of HIF1α target genes [11] while nuclear translocated-PKM2 following EGF stimulation acts as a β-catenin coactivator. The resulting downstream induction of gene expression promotes the Warburg effect, namely the preferentially weighting of cancer cells towards glycolysis, even in the presence of sufficient oxygen tension [12].

Different long noncoding RNAs (lncRNAs) have been found to be integral regulators of glycolysis, acting in both positive and negative manners, either through direct effects on glycolytic enzymes or indirectly through interconnected pathways. For example, previously we showed that lincRNA-21 promotes HIF1α stabilization to enhance glycolysis under hypoxic conditions [13] whereas IDH1-AS1-mediated dimerization of IDH1 increases α-KG and decreases ROS levels to down-regulate HIF1α and inhibit glycolysis [14]. And more recently, we showed that a metabolon formed by glycoLINC engages all lower glycolytic enzymes (inclusive of PKM2) to support glycolytic efficiency under nutritional stress [15]. In an intriguingly similar manner, a recent study reported that HULC acts as an adaptor to promote binding between LDHA, PKM2 and FGFR1 to enhance glycolysis [16]. Furthermore, glycolysis is also promoted by FEZF1-AS1 binding to and stabilizing PKM2 [17] while LINC00689 sponging of miR-338-3p promotes PKM2 expression to increase glycolysis in glioma cells, resulting in elevated cell growth and metastasis [18]. However, it can be noted that none of the presently reported examples of lncRNA-mediated regulation of PKM2 involve its nuclear functions. Regarding this point, we now demonstrate that the lncRNA MNX1-AS1, also known as CCAT5, plays an essential role in regulating the non-glycolytic nuclear function of PKM2 to promote the Warburg effect in hepatocellular carcinoma (HCC).

MNX1-AS1 is a pan-cancer lncRNA that expressed in a variety of cancers including colorectal cancer, cervical cancer, ovarian cancer, prostate cancer, breast cancer, hepatocellular carcinoma and intrahepatic cholangiocarcinoma [19–26]. Elevated expression of MNX1-AS1 plays multiple functions in tumorigenesis. For example, MNX1-AS1 facilitates COMMD8 expression by sponging miR-218-5p, and then promotes hepatocellular carcinoma progression [19]. Moreover, MNX1-AS1 was found to be activated by c-Myc and stabilized YB1, resulted in progression of colorectal cancer [26]. Intriguingly, a recent report identified nine glycolysis-related lncRNAs have prognostic significance in bladder cancer by bioinformatics analysis. Notably, MNX1-AS1 was one of these glycolysis-related lncRNAs and considered as a risk factor for the prognosis of bladder cancer [27]. However, the precise role and detail mechanism of MNX1-AS1 on aerobic glycolysis are largely unknown. We show that MNX1-AS1 is transcriptionally activated by c-Myc in response to EGF stimulation. Mechanistically, MNX1-AS1 serves as a scaffold to facilitate interactions between PKM2 and importin α5 with this complex permitting the translocation of PKM2 into the nucleus. In turn, the accumulation of nucleus PKM2 led to the elevated expression of key glycolysis-associated genes, thereby enhancing the Warburg effect. Manipulating the levels of MNX1-AS1 resulted in marked changes in glycolytic flux, impacting the proliferation of HCC cells both in vitro and in vivo. Moreover, the frequent upregulation MNX1-AS1 observed in HCC tissues suggests that targeting MNX1-AS1 offers new therapeutic opportunities in HCC.

## Methods

### Cell culture

The cell lines HEK293T, HepG2, SK-Hep-1,Hep3B, PLC/PRF/5, HCCLM3, Huh7, WRL68, HAFF cells were maintained in DMEM (Gibco) supplemented with 1% sodium pyruvate, 1% penicillin/streptomycin and 10% FBS at 37 °C with 5% CO_2_. SMMC-7721 was maintained in RPMI-1640 supplemented with1% sodium pyruvate, 1% penicillin/streptomycin and 10% FBS at 37 °C with 5% CO_2_.

### Human tissue cDNA and sections

Human tissue cDNA and sections were obtained from Shanghai Outdo Biotech Co., Ltd. (Shanghai, P. R. China). The study is compliant with all relevant ethical regulations for human research participants, and all participants provided written informed consent.

### RNA interference

HEK293T cells were transfected with shRNAs (cloned in PLKO.1), pREV, pGag and pVSVG plasmids at the ratio of 2:2:2:1. Twenty-four hours after transfection, cells were changed to fresh medium for an additional 24 h. The medium supernatant containing lentiviral was filtered by 0.45 μm filter and used for infection. shRNA sequences are shown in Supplementary Table S4.

### RNA extraction

Total RNA were isolated using the TRIzol reagent (Invitrogen, AM9738). Then followed by DNase-treatment. RNA was subsequently purified by phenol-chloroform extraction. Then subjected to Northern blot, qPCR or semi-quantitative RT-PCR.

### QPCR & semi-quantitative RT-PCR

Total RNA was isolated using TRIzol. 1 μg of RNA was used to synthesize cDNA using Takara RT reagent kit (RR036A) according to the manufacturer’s instruction. Real-time PCR was performed using SYBR Green real-time PCR analysis (RR820A). Threshold cycle numbers (ct value) were normalized against an internal control (β-actin), then figured out the relative folds. Semi-quantitative RT-PCR was performed using 2xTaq PCR mix (Vazyme Biotech), the PCR reaction was carried out in a programmable thermal cycler with indicated primer pairs (Supplementary Table S4). The PCR reactions were cycled by appropriate cycles (25 cycles for internal control, 30–40 cycles for MNX1-AS1).

### In vitro transcription

In vitro transcription was performed using the T7-Flash Biotin RNA Transcription Kit (Epicentre ASB71110, for biotin label) or TranscriptAid T7 High Yield Transcription Kit (Thermo Scientific #K0441, without biotin label) according to the manufacturer’s instructions.

### Northern blot

Northern blot analysis was performed by DIG Northern Starter Kit (Roche, 12,039,672,910). 10 μg of total RNA was loaded on a 1% denaturing agarose gel and run for 2–2.5 h, then transferred to Hybond-N membrane (GE). Membranes were dried and ultraviolet-crosslinked (1x at 200,000 μJ/cm^2^ at 265 nm). Pre-hybridized the membrane at 55 °C for 1 h and hybridized the membrane with digoxin-labelled RNA probes at 55 °C overnight. For data collection, anti-digoxin-AP was used to visualize the blot by enhanced chemiluminescence (Tanon 5200).

### Western blot and immunoprecipitation

Western Blot and Immunoprecipitation assays were performed as previously described [28]. Antibodies and dilutions used are shown in the Supplementary Table S4.

### Lactate and glucose uptake measurement

Cellular lactate and glucose levels was measured by lactate assay kit (BioVision, K607) and Glucose Assay Kit (BioVision, K606) according to the manufacturer’s instructions, respectively.

### ECAR rate measurement

Assays were performed using the Seahorse XFe96 analyzer (Seahorse Bioscience, Agilent) according to the manufacturer’s instructions. Briefly, 1–2 × 10^4^ cells/well were seeded in a 96-well 24 h before assay. ECAR was measured in XF base medium (pH 7.4) supplemented with 1 mM glutamine, glucose (10 mM), oligomycin (1 mM) and 2-DG (50 mM).

### Cytosolic/nuclear fractionation

Cells were treated with hypotonic buffer (25 mM Tris–HCl (pH 7.4), 1 mM MgCl2 and 5 mM KCl) for 5 min on ice. NP-40 was then added to the buffer at a final concentration of 0.5%, and the sample was left on ice for another 5 min. The supernatant was collected as the cytosolic fraction after centrifugation at 5000 g for 5 min. Resuspended the pellets in resuspension buffer (20 mM HEPES (pH 7.9), 400 mM NaCl, 1 mM dithiothreitol, 1 mM EGTA, 1 mM EDTA and 1 mM phenylmethyl sulfonyl fluoride). The nuclear fraction was collected by centrifugation at 12,000 g for 5 min.

### In situ hybridization

ISH was carried out as previously described [29]. FFPE sections were treated with 10% hydrogen peroxide and pepsin (2 μg per ml, EXIQON) for 1 h at 37 °C and then deparaffinized. Sections were then incubated with hybridization solution with MNX1-AS1 probes (In vitro transcription synthesized with digoxigenin (DIG)-labelled) at 42 °C for 24 h. Then washed with 5 × SSC (Gibco) for 10 min, followed by washing with 2 × SSC for 10 min and 0.2 × SSC at 55 °C for 10 min. Sections were blocked with the blocking buffer (PBST with 5% BSA and 10% normal goat serum) at 37 °C for 1 h. The anti-DIG antibody and horseradish peroxidase-coupled second antibody were used for DAB chromogenic. Counterstaining was carried out using haematoxylin before dehydration. The intensity of staining was judged by IHC profiler in ImageJ software.

### RNA FISH and immunofluorescence staining

In situ hybridization was performed using specific RNA probes target to MNX1-AS1. RNA probes were synthesized by In vitro transcription of T7 template. 1 μg RNA was labelled with Alexa Fluor® 546 dye by ULYSIS® Nucleic Acid Labeling Kits (thermofisher, U21652). Cells were fixed with methanol and acetic acid (3:1) for 10 min, and then permeabilized with 0.2% Triton X-100 for 10 min. After prehybridization (1 × PBS/0.5% Triton X-100), cells were hybridized in hybridization buffer (10% Dextran sulfate, 50% formamide, 2 × SSC, 1xDenhardt’s solution, 10 mM EDTA(pH 8.0), 1 mg/ml yeast transfer RNA, 1 mg/ml sheared salmon sperm DNA) with MNX1-AS1 probes at 55 °C overnight. For the following IF staining, cells were washed thrice before blocking with 5% BSA for 1 h, then incubated with indicated primary antibodies and fluorescence-labeled secondary antibodies. Nuclei were stained with 1 μg/ml DAPI for 1 min. Images were visualized by Laser confocal microscopy (Zeiss LSM880).

### Recombinant protein purification

His-tagged importin α5 and GST-tagged PKM2 were expressed in *E.coli* bacteria. Proteins were purified using Ni-NTA Sepharose (Beyotime, P2226) and glutathione Sepharose (Beyotime, P2262), followed by dialyzed against 10% glycerol and 20 mM Tris-HCl (pH 8.0) at 4 °C overnight.

### Biotin RNA pulldown assay

RNA pulldown assays were performed as previously described [28]. Briefly, cells were lysed in RIP buffer (25 mM Tris (pH 7.4), 150 mM KCl, 0.5 mM dithiothreitol, 0.5% NP-40, with RNase inhibitors and protease inhibitors cocktail) and followed by pre-cleared against empty beads. Biotin-labelled RNA or biotin-labelled DNA probes were incubated with streptavidin magnetic beads, and then incubated with cell lysate at 4 °C for 4 h before washing five times in RIP buffer. Binding protein and RNA were eluted in Laemmli sample buffer.

### Electrophoretic mobility shift assay

The biotin labelled MNX1-AS1 RNA (2 nM) was mixed in the binding reaction system with recombinant forms of His-tagged importin α5 (1 μg) or GST-tagged PKM2 (1 μg). Unlabeled cyclized MNX1-AS1 (10 μM) was used as competitor. Reactions were subjected to gel electrophoresis and transferred to nylon membranes. The bands were detected using the streptavidin-HRP conjugate.

### Luciferase reporter and mammalian two-hybrid assays

Luciferase Reporter Assays were performed according to the manufacturer’s instructions (Promega). The Mammalian two-hybrid assay was performed as previously described [28]. In brief, PKM2 and importin α5 DNA sequences were cloned into the pBIND and pACT vectors as indicated to generate fusion proteins with the DNA-binding domain of GAL4 and the activation domain of VP16. These constructs were transfected along with the pG5luc vector into cells with stable knockdown of MNX1-AS1. 48 h later, luciferase activity was measured by the Dual-Luciferase Reporter Assay System.

### Absolute RNA quantitation

Absolute RNA quantitation was performed using Clarity ^(^™^)^ digital PCR system. cDNA preparation was carried out using qScript cDNA SuperMix for MNX1-AS1 with DNase treated RNA. MNX1-AS1 was amplified by PCR with divergent primers for 40 cycles. The annealing temperature was 58 °C. The data were analyzed with the Clarity™ software are given as copies per μl and were converted to copies per cell based on the known cell equivalents of input cDNA.

### Chromatin immunoprecipitation assays

Chromatin immunoprecipitation assays were performed by using c-Myc antibodies (CST, 18583) and the Millipore ChIP kit (17-371RF) according to the manufacturer’s instructions. Semi-quantitative RT PCR primers used are shown in the Supplementary Table S4.

### Colony formation assay

HepG2 cells (1 × 10^3^) with and without stable knockdown of MNX1-AS1 or Huh7 cells (1 × 10^3^) stably expressing pCDH or pCDH-MNX1-AS1 were cultured in a 6-well plate. 14 days later, cells were stained with crystal violet before fixed, and then photographed.

### Xenograft mouse model

The Balb/c nude mice (4 weeks old, ♂) were obtained from Shanghai SLAC Laboratory Animal Co.,Ltd. and housed in a specific pathogen free facility. PLC/PRF/5 (5 × 10^6^) cells with stable knockdown of MNX1-AS1 or Huh7 (2 × 10^6^) cells overexpression of MNX1-AS1, and indicated ctrl cells were injected subcutaneously into the dorsal flanks of nude mice. 28 days after injection, mice were sacrificed and tumors were excised and weighed. Studies on animals were conducted with approval from the Animal Research Ethics Committee of University of Science and Technology of China.

### Statistics and reproducibility

All the data were repeated at least three times. Statistical analysis was carried out using Microsoft Excel software and GraphPad Prism to assess the differences between experimental groups. Data were analysed by two-tailed Student’s t-test for comparions of two samples, one-way ANOVA with Tukey’s post-test for univariate comparisons, two-way ANOVA with Bonferroni’s post-test for bivariate comparisons. *P* < 0.05 were considered to be statistical significance. *p < 0.05, **p < 0.01, and ***p < 0.001.

## Results

### MNX1-AS1 is a c-Myc associated pan-cancer lncRNA

**Fig. 1:**
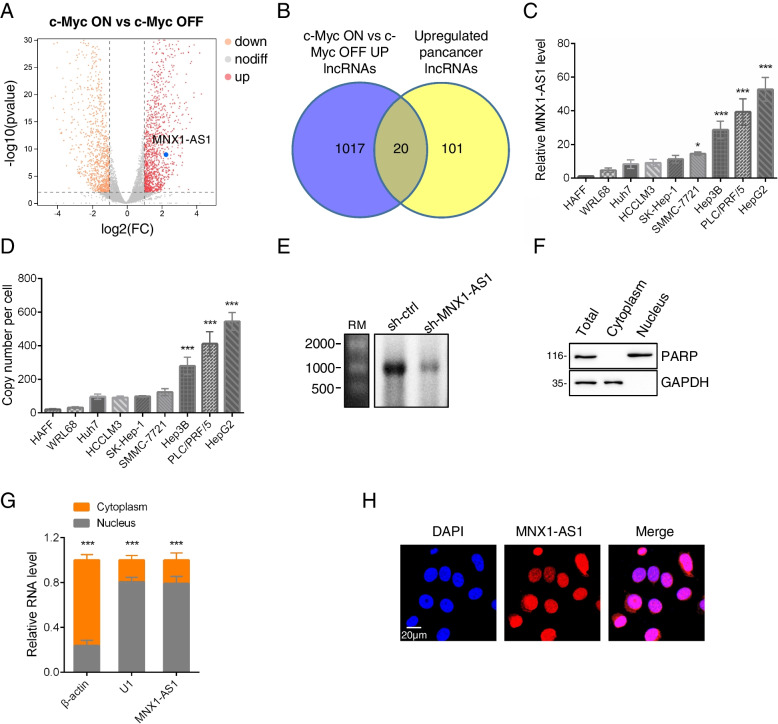
MNX1-AS1 is a c-Myc associated pan-cancer lncRNA. **a** Volcano Plot showing differentially expressed lncRNAs based on RNA-seq analysis of P493–6 cells carrying a Tet-Off c-Myc expression system following the induction of c-Myc with 1 μM doxycycline. MNX1-AS1 is highlighted in blue. **b** Venn diagram showing the intersection of c-Myc-upregulated lncRNAs from (**A**) versus pan-cancer lncRNAs identified from the TCGA database. **c** qPCR assays measuring MNX1-AS1 expression in hepatocellular carcinoma and normal cell lines. **d** Absolute quantitation of MNX1-AS1 by digital PCR in hepatocellular carcinoma and normal cell lines. **e** Northern blotting against MNX1-AS1 comparing HepG2 cells bearing sh-ctrl or sh-MNX1-AS1 lentiviruses. **f, g** Fractionation of HepG2 cells to determine the subcellular localization of MNX1-AS1. Western blotting against control proteins PARP and GAPDH (**F**) and qPCR against MNX1-AS1, β-actin and U1 control RNAs (**G**) in cytoplasmic and nuclear fractions, respectively. **h** Fluorescence in situ hybridization in HepG2 cells against MNX1-AS1 (red) with cell nuclei decorated by DAPI (blue). **C** and **D** are mean ± SD; *n* = 3 independent experiments, one-way ANOVA with Tukey’s multiple comparison post-test, *p < 0.05, ***p < 0.001. **G** is mean ± SD; *n* = 3 independent experiments, two-tailed paired Student’s t test, ***p < 0.001. **E**, **F** and **H** are represent of three independent experiments

The protooncogene c-Myc is considered a master regulator of tumorigenesis. On this basis, we sought to uncover lncRNAs that are involved in mediating the downstream functions of c-Myc. To identify lncRNAs that are responsive to c-Myc, we took advantage of P493–6 human lymphoblastoid cells bearing a conditional c-Myc tet-off system. Using array profiling, a total of 1037 validated lncRNAs were revealed to be positively associated with c-Myc expression (Fig. 1A, Supplementary Table S1). To further rationalize these data, we further considered which lncRNAs were commonly upregulated in diverse cancer types by interrogating the 20 most common tumor types in the TCGA database. This analysis uncovered 121 pan-cancer lncRNAs that were upregulated in diverse tumor types with subsequent Venn analysis revealing 20 pan-cancer lncRNAs in common between the c-Myc-upregulated lncRNAs (Fig. 1B, Supplementary Table S2). Among these 20 c-Myc associated pan-cancer lncRNAs, MNX1-AS1 attracted our attention because of prior links established with c-Myc although there was no clear consensus regarding its cancer-related functions (refer Discussion). Thus, we focused our efforts towards understanding the contribution of MNX1-AS1 to cancer cells.

MNX1-AS1 (LOC645249) is the antisense lncRNA proximal to the MNX1 gene located on chromosome (chr) 7 and contains two exons with a predicted transcript length of 1041 nt (Fig. S1A). In order to further analyze MNX1-AS1 function it was first important to establish appropriate cell line models. Towards this, we assessed the expression levels of MNX1-AS1 in a panel of seven hepatocellular carcinoma (HCC) derived lines. QPCR analysis revealed that MNX1-AS1 expression varied in different HCC cell lines but with generally higher expression compared to the normal hepatic cell lines, HAFF and WRL68 (Fig. 1C). Consistently, comparison of absolute transcript levels by digital PCR between these seven HCC cells and two normal cells showed similar results (Fig. 1D). Moreover, we found that c-Myc expression was positively correlated with MNX1-AS1 levels in most of normal and liver cancer cell lines (Fig. S1B). Further characterization using Northern blotting of HepG2 cells revealed a major ~ 1000 nt band, consistent with the annotated transcript from the UCSC Genome Browser (Fig. 1E). Notably, silencing of MNX1-AS1 attenuated the signal intensity of the reactive band, indicating this represented the bona fide MNX1-AS1 transcripts. Lastly, we considered where MNX1-AS1 is located within cells. Parallel experiments using cytosolic/nuclear fractionation and FISH showed that MNX1-AS1 was mainly expressed in the cell nucleus (Fig. 1F-H).

### MNX1-AS1 is a direct target of c-Myc

**Fig. 2:**
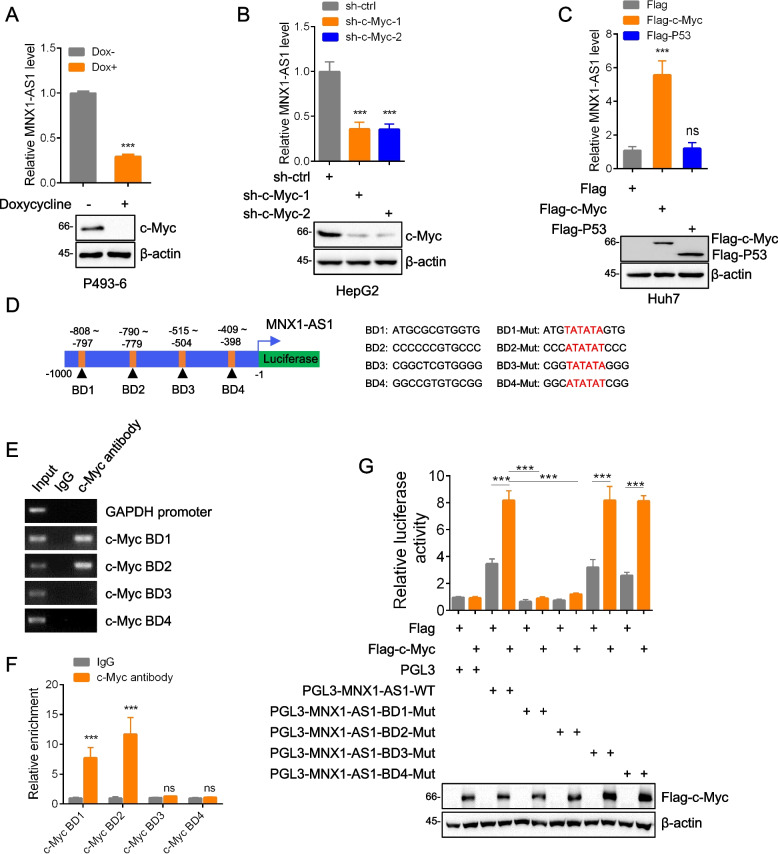
MNX1-AS1 is a direct target of c-Myc. **a** P493–6 cells carrying a Tet-Off c-Myc expression system treated with 1 μM doxycycline were subjected to qPCR and Western blot analysis, respectively. **b** and **c** MNX1-AS1 levels were measured by qPCR after infection of HepG2 cells with lentiviruses containing control shRNA (sh-ctrl) or two independent shRNAs targeting c-Myc (**B**), or after transfection of Huh7 cells with either empty Flag (control), Flag-c-Myc or Flag-P53 (negative control) plasmids (**C**). **d** Schematic of the putative c-Myc binding domains (BD) in the proximal promoter of MNX1-AS1 illustrating the design of mutant constructs as luciferase reporters. **e** and **f** ChIP assays were conducted in HepG2 cells using c-Myc antibodies or control IgG. Bound DNA fragments were subjected to semi-quantitative RT-PCR (**E**) or qPCR (**F**) using the specified primers. **g** Luciferase reporter assays in HepG2 cells after co-transfection of the plasmids from (**D**) for 24 h. The expression of c-Myc was confirmed by Western blotting. **A** is mean ± SD; *n* = 3 independent experiments, two-tailed paired Student’s t test, ***p < 0.001. **B**, **C** and **G** are mean ± SD; *n* = 3 independent experiments, one-way ANOVA with Tukey’s multiple comparison post-test, ns, not significant, ***p < 0.001. **E** is represent of three independent experiments. **F** is mean ± SD; n = 3 independent experiments, two-way ANOVA with Bonferroni’s multiple comparison post-test, ns, not significant, ***p < 0.001

We next sought to determine the basis of why MNX1-AS1 expression was correlated with c-Myc. As anticipated, analysis of P493–6 cells with inducible c-Myc turn-off showed that MNX1-AS1 expression tracked with the levels of c-Myc manipulated by doxycycline treatment (Fig. 2A). We then expanded these experiments to include knockdown of c-Myc in HCC cell lines with high MNX1-AS1 expression (HepG2 and PLC/PRF/5) and overexpresssion of c-Myc in low MNX1-AS1 expressing lines (Huh7 and HCCLM3). Consistent with the results in P493–6 cells, we observed that c-Myc knockdown reduced MNX1-AS1 levels while enforced c-Myc expression resulted in the opposite effects (Fig. 2B, C and S2A-S2D). Importantly, the use of two independent shRNA sequences to target c-Myc produced identical responses, indicative that our findings were not related to off-target effects. Moreover, we also introduced Flag-P53 as a negative control, and found that Flag-P53 has no contribution to MNX1-AS1 levels. Collectively, these data establish that MNX1-AS1 levels are positively correlated with those of c-Myc.

As c-Myc is fundamentally a transcription factor, the preceding data suggested that c-Myc may be directly responsible for the transactivation of MNX1-AS1. Using the JASPAR database, we identified four putative c-Myc binding sites within 1000 bp upstream of the *MNX1-AS1* transcriptional start site (Fig. 2D). We designated these binding domains (BD) 1–4 and using chromatin immunoprecipitation (ChIP) assays observed that BD1 and BD2 but not BD3 and BD4 were responsible for c-Myc binding to the *MNX1-AS1* promoter (Fig. 2E and F). Next, to determine the function of c-Myc binding at these sites, we prepared luciferase reporters enclosing the *MNX1-AS1* proximal promoter, both unaltered and with the individual BD sequences inactivated by mutation. Notably, the luciferase reporter activity of the wildtype construct was increased after ectopic expression of c-Myc whereas mutation of BD1 or BD2 abolished the transcriptional increases resulting from c-Myc overexpression (Fig. 2G). Moreover, consistent with the ChIP assays, mutation of BD3 or BD4 was unable to influence luciferase activity. Notably, similar to qPCR analysis previously, overexpression of Flag-P53 failed to induce luciferase activity of wildtype reporter (Fig. S2E). Together these data clearly establish that MNX1-AS1 is transcriptionally regulated by c-Myc with its occupancy of the *MNX1-AS1* promoter requires both BD1 and BD2 with sites.

### MNX1-AS1 promotes glycolysis in HCC

**Fig. 3:**
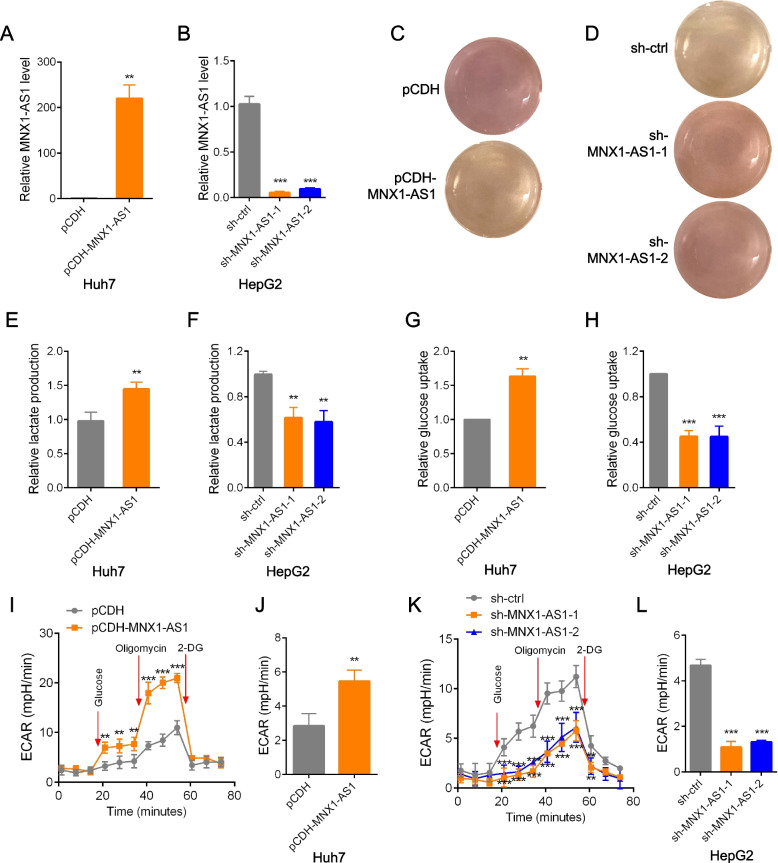
MNX1-AS1 promotes glycolysis in HCC. **a** and **b** MNX1-AS1 levels were measured by qPCR after infection of Huh7 cells with either pCDH control or pCDH-MNX1-AS1 lentiviruses (**A**), or after infection of HepG2 cells with lentiviruses containing control shRNA (sh-ctrl) or two independent shRNAs targeting MNX1-AS1 (**B**). **c** and **d** Huh7 (**C**) and HepG2 (**D**) cells from (**A**) and (**B**) were seeded in 12 wells and cultured in DMEM for 12 hr. Acidification of the culture medium was evaluated by visual inspection. **e** and **f** Extracellular lactate production in Huh7 (**E**) and HepG2 (**F**) cells from (A) and (B). (**g** and **h**) Glucose uptake were measured in Huh7 (**G**) and HepG2 (**H**) cells from (**A**) and (**B**). **i**-**l **ECAR measurements using Seahorse XF assays in Huh7 (**I** and **J**) and HepG2 (**K** and **L**) cells from (**A**) and (**B**). The glycolysis rate were calculated as: (Maximum ECAR before Oligomycin injection)-(Last ECAR before Glucose injection) (**J**) and (**L**). **A**, **E**, **G** and **J** are mean ± SD; *n* = 3 independent experiments, two-tailed paired Student’s t test, **p < 0.01. **B**, **F**, **H** and **L** are mean ± SD; *n* = 3 independent experiments, one-way ANOVA with Tukey’s multiple comparison post-test, **p < 0.01, ***p < 0.001. **C** and **D** are represent of three independent experiments. **I** and **K** are mean ± SD; *n* = 3 independent experiments, two-way ANOVA with Bonferroni’s multiple comparison post-test, ns, not significant, **p < 0.01, ***p < 0.001

To better understand the functional role of MNX1-AS1, we next determined if manipulating its levels would affect growth phenotypes in HCC cell lines. As before, we selected the Huh7 and HCCLM3 cell lines with low MNX1-AS1 expression for overexpression studies and the HepG2 and PLC/PRF/5 cell lines for knockdown experiments. The efficacy of these manipulations was verified using QPCR analysis (Fig. 3A, B, S3A and S3B). Casual examination of the cultures revealed that enforced expression of MNX1-AS1 in Huh7 cells significantly accelerated culture acidification (Fig. 3C), even though the growth rate of Huh7 cells remained practically unaltered. Conversely, silencing of MNX1-AS1 in HepG2 cells led to reduced culture medium acidification (Fig. 3D). Together, these data indicate that MNX1-AS1 promotes culture media acidification, a phenomenon generally associated with increases in glycolytic activity which results from pH changes due to secreted lactate.

To test this notion, we measured extracellular lactate levels after manipulating MNX1-AS1 expression. As anticipated, ectopic expression of MNX1-AS1 in Huh7 and HCCLM3 cells increased secreted lactate levels while knockdown of MNX1-AS1 in HepG2 and PLC/PRF/5 cells decreased lactate levels (Fig. 3E, F, S3C and S3D). Furthermore, consistent with the conclusion that altering MNX1-AS1 expression affects glycolysis, we found there were remarkably similar changes in glucose uptake where the overexpression or silencing of MNX1-AS1 resulted in elevated and decreased glucose uptake, respectively (Fig. 3G, H, S3E and S3F). As definitive evidence of these effects, we lastly performed glycolytic stress tests measuring the extracellular acidification rate (ECAR), a proxy measure of glycolytic flux. Indeed, we observed striking differences in the assay profiles where overexpression of MNX1-AS1 significantly increased ECAR in Huh7 and HCCLM3 cells (Fig. 3I, J, S3G and S3H). Conversely, knockdown of MNX1-AS1 significantly reduced ECAR in HepG2 and PLC/PRF/5 cells (Fig. 3K, L, S3I and S3J). Collectively, these data indicate that MNX1-AS1 functions to increase glucose uptake while promoting increases in aerobic glycolysis.

### MNX1-AS1 directly binds to PKM2 and importin α5

**Fig. 4:**
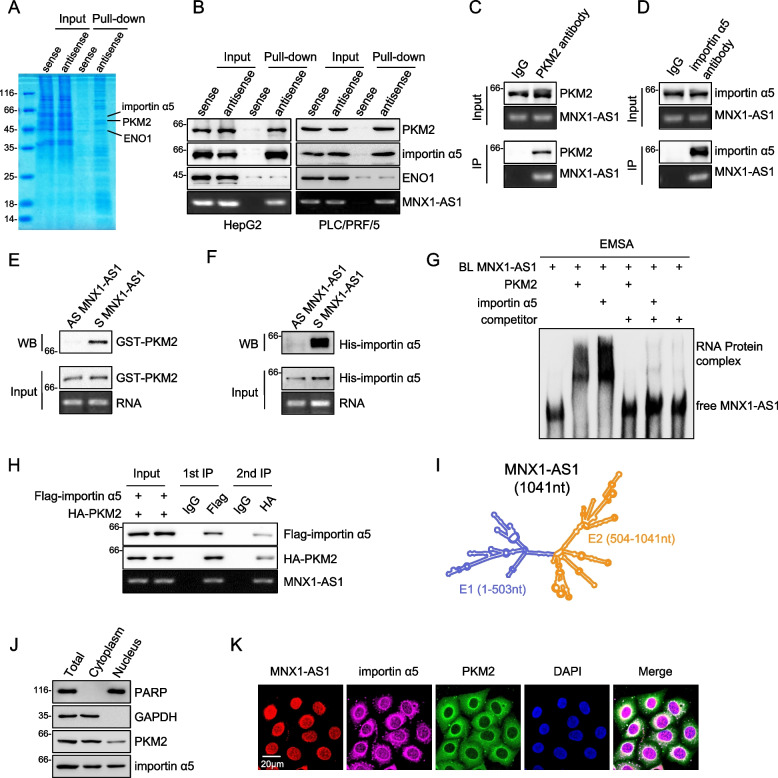
MNX1-AS1 directly binds to PKM2 and importin α5. **a** RNA protein pull-down assays using biotin-labelled sense or anti-sense MNX1-AS1 probes against cell lysates from HepG2 cells. PKM2 and importin α5 were subsequently identified by using mass spectrometry. **b** Biotin-labeled sense (S) or antisense (AS) MNX1-AS1 probes were used for RNA-protein pull-down against HepG2 (left) and PLC/PRF/5 (right) cell lysates. **c** and **d** RIP assays conducted in HepG2 cells against PKM2 (**C**) or importin α5 (**D**) with samples analyzed by Western blotting and semi-quantitative RT-PCR against MNX1-AS1. **e** and **f** RNA pull-down assays were conducted with biotin-labelled sense or anti-sense MNX1-AS1 adsorbed to streptavidin-conjugated beads against the indicated GST-tagged-PKM2 or His-tagged-importin α5 proteins purified from *E.coli*. Results were assessed by Western blotting. **g** Electrophoretic Mobility-Shift Assays (EMSA) analysis of the interactions between biotin-labelled MNX1-AS1 (2 nM) and recombinant GST-tagged-PKM2 or His-tagged-importin α5 proteins. Signals were revealed by Streptavidin-HRP. Unlabeled MNX1-AS1 (10 μM) was used as competitor. **h** HepG2 cells co-transfected with FLAG-importin α5 and HA-PKM2 were used to perform sequential immunoprecipitations with anti-FLAG and anti-HA antibodies. **i** Structure plot of MNX1-AS1 based on minimum free energy predicted by Vienna RNA. Exon1 (E1, nt 1–503, blue) and exon 2 (E2, nt 504–1041, orange) are color highlighted. **j** Subcellular fractions of HepG2 cells were analyzed by Western blotting to determine the subcellular localization of PKM2 and importin α5. **k** Fluorescence in situ hybridization of MNX1-AS1 followed by immunofluorescence staining of PKM2 and importin α5 in HepG2 cells. Cell nuclei were decorated by DAPI (**A**-**H**, **J** and **K**) are represent of three independent experiments

Given our finding above that MNX1-AS1 primarily exists within the nucleus, we were intrigued as to how MNX1-AS1 functioned to regulate glycolysis. To glean further clues, we used RNA pulldown assays combined with mass spectrometry to investigate the MNX1-AS1 protein interactome. Surprisingly, within the shortlist of candidate proteins we detected the glycolytic enzymes PKM2 and ENO1 (Fig. 4A, S4A and Supplementary Table S3). Using Western blotting as the readout, we confirmed that selective interactions occurred between MNX1-AS1 and PKM2, while ENO1 appeared to be nonspecifically captured in the RNA pull-down assays (Fig. 4B). A further notable candidate identified by mass spectrometry was KPNA1 (also known as importin α5), previously reported as a PKM2 binding protein associated with the regulation of PKM2 nuclear translocation [12]. Indeed, we verified that importin α5 was robustly recovered with MNX1-AS1 (Fig. 4B and S4A). To confirm these data, we next performed reciprocal assays using RNA immunoprecipitation (RIP) against PKM2 and importin α5. Consistent with the RNA pulldown assay results, MNX1-AS1 was readily co-precipitated with endogenous PKM2 and importin α5 (Fig. 4C and D). Together, these results showed that MNX1-AS1 can bind PKM2 and importin α5. However, it was not clear if these interactions occurred discretely or as part of a complex, or perhaps even required other intermediary proteins.

To resolve these possibilities, we reconstructed interactions between MNX1-AS1, PKM2 and importin α5 using recombinant proteins purified from bacteria. Accordingly, GST-PKM2 and His-importin α5 were individually mixed with in vitro transcribed biotin-labelled sense or anti-sense MNX1-AS1 followed by capture with streptavidin magnetic beads and Western blotting. This analysis conclusively showed that PKM2 and importin α5 were both able to bind independently to MNX1-AS1 in a direct manner (Fig. 4E and F). Further substantiating these findings, electrophoretic mobility shift assays (EMSA) confirm that supershifts occurred when MNX1-AS1 was incubated with PKM2 or importin α5, respectively (Fig. 4G). We then asked whether MNX1-AS1 also forms a trimeric complex with PKM2 and importin α5 using a two-step immunoprecipitation method. After co-transfecting cells with Flag-importin α5 and HA-PKM2, lysates were first immunoprecipitated with anti-Flag antibodies before elution and secondary precipitation against HA. Instructively, Flag-importin α5, HA-PKM2 and MNX1-AS1 were all captured in the first and second phase immunoprecipitations, providing clear evidence that MNX1-AS1 forms a ternary complex with PKM2 and importin α5 (Fig. 4H).

To further delineate the structural determinants of interactions between MNX1-AS1, PKM2 and importin α5, we next undertook domain mapping experiments. According to minimum free energy modelling, the secondary structure of MNX1-AS1 is bipolar with each of the two exons contributing to one pole of the molecule. Thus, we divided MNX1-AS1 into two subfragments delineated by exons, namely E1 (1–503 nt) and E2 (504–1041 nt) (Fig. 4I). RIP analyses revealed that the E2 fragment was able to bind to GST-PKM2, while the E1 fragment interacted with His-importin α5 (Fig. S4B and S4C). In concert, we then sought to establish which domains in PKM2 and importin α5 were required for binding with MNX1-AS1. Based on the protein domain annotations in UniProtKB, we subdivided PKM2 and importin α5 each into four fragments and cloned these into p3xFlag vectors to enable expression (Fig. S4D and S4E). As expected, RIP assays demonstrated that full length PKM2 and importin α5 were capable of binding to MNX1-AS1 with assessment of the truncated proteins showing that MNX1-AS1 interacted with the P4 subfragment of PKM2 and the P2 subfragment of importin α5, respectively (Fig. S4D and S4E). Thus, distinct domains in MNX1-AS1 are responsible for interactions with PKM2 and importin α5. And intriguingly, the MNX1-AS1-binding regions in PKM2 and importin α5 proteins contain their known nuclear localization signals (NLS).

Lastly, we investigated the cellular context of the interaction between MNX1-AS1, PKM2 and importin α5. We first conducted a broad survey of PKM2 and importin α5 levels after overexpression or knockdown of MNX1-AS1 in different HCC cell lines. Nonetheless, manipulating MNX1-AS1 levels failed to alter PKM2 or importin α5 expression (Fig. S4F), indicative that MNX1-AS1 does not influence the expression of PKM2 and importin α5. We then considered where the ternary complex is located within cells. Notably, cytosolic/nuclear fractionation assays showed that importin α5 was found in the cytoplasm and nucleus while PKM2 was predominantly located in the cytoplasm with a small but obvious pool in the nucleus (Fig. 4J). Additional combined imaging of fluorescence in situ hybridization (FISH) and immunofluorescence (IF) staining decorating MNX1-AS1, PKM2 and importin α5 showed that co-localization signals between all markers occurred mainly on the surface of the nuclear membrane (Fig. 4K).

Based on these collective findings, we considered the possibility that MNX1-AS1 was involved in regulating the import of PKM2 into the nucleus.

### MNX1-AS1 enhances PKM2 nuclear translocation in response to EGF stimulation

Previous work revealed PKM2 is translocated to the nucleus in human cancer cells in response to EGF, a process resulting from PKM2 successively binding to PIN1 (peptidylprolyl cis/trans isomerase, NIMA-interacting 1) and importin α5 [12]. As noted above, we found MNX1-AS1 binds with both PKM2 and importin α5, although we did not identify PIN1 in the mass spectrometry screen (Supplementary Table S3). Consistently, Western blotting showed that MNX1-AS1 did not associate with PIN1 (Fig. S5A). We then extended our investigations to uncover the specific role of MNX1-AS1 in the ternary complex.

**Fig. 5:**
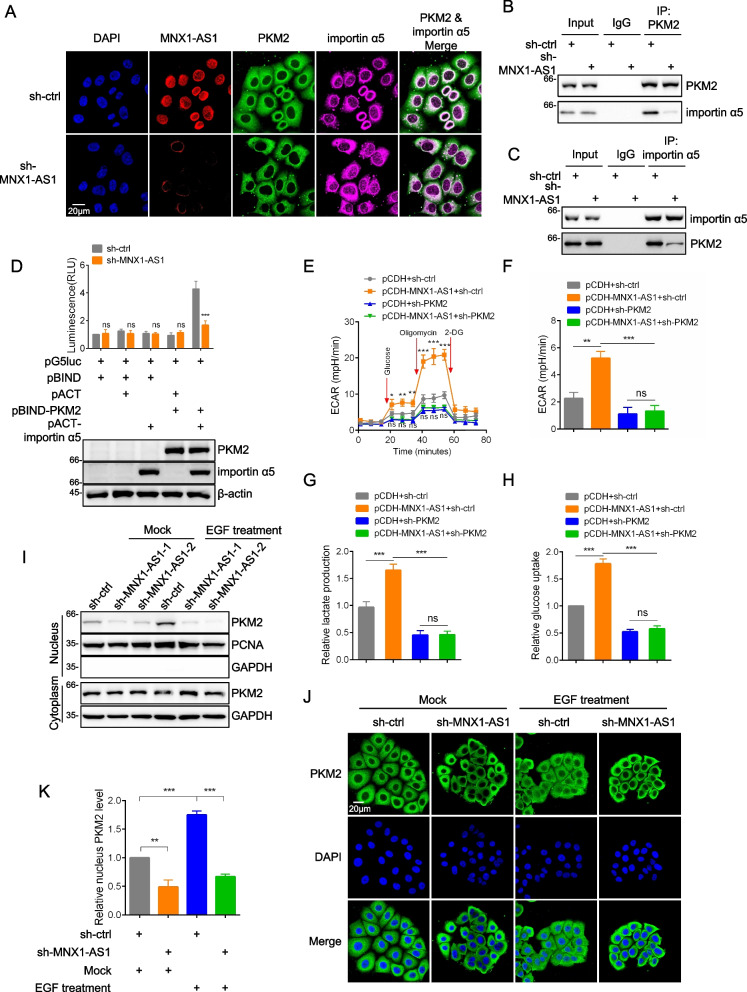
MNX1-AS1 enhances PKM2 nuclear translocation in response to EGF. **a** HepG2 cells were infected with lentiviruses containing control shRNA (sh-ctrl) or shRNA targeting MNX1-AS1. Fluorescence in situ hybridization of MNX1-AS1 followed by immunofluorescence staining of PKM2 and importin α5. Cell nuclei were decorated by DAPI. **b** and **c** Co-immunoprecipitation analysis conducted in HepG2 cells bearing either sh-ctrl or sh-MNX1-AS1 lentiviruses. Cell lysates were immunoprecipitated by antibodies against PKM2 (**C**) or importin α5 (**D**) with potential interactions determined by Western blotting. **d** Mammalian two-hybrid assays were used to measure interactions between PKM2 and importin α5 in the presence and absence of endogenous MNX1-AS1. The indicated combinations of pBIND/pACT plasmids were transfected into HepG2 cells stably expressing either sh-ctrl or sh-MNX1-AS1 before measuring relative luciferase activity (top; a proxy for the interaction between proteins). Western blotting verified the presence of the transfected fusion proteins (bottom). **e**-**h** Huh7 cells infected with sh-ctrl or sh-PKM2 lentiviruses were subjected to infection of pCDH control or pCDH-MNX1-AS1 lentiviruses. ECAR measurements using Seahorse XF assays (**E** and **F**), Extracellular lactate production (**G**) and Glucose uptake (**H**) were measured. The glycolysis rate were calculated as: (Maximum ECAR before Oligomycin injection)-(Last ECAR before Glucose injection). **i**-**k** HepG2 cells were infected with lentiviruses containing sh-ctrl or sh-MNX1-AS1. Cells were with and without EGF (100 ng/ml) treatment before subcellular fractions. Nucleus PKM2 were assessed by Western blot analysis (**I**). Cells from (**I**) were subsequently subjected to immunofluorescence staining of PKM2 (**J**), and nucleus PKM2 was measured using ZEN imaging software (K). **A**, **B**, **C**, **I** and **J** are represent of three independent experiments. **D**-**H** and **K** are mean ± SD; *n* = 3 independent experiments, two-way ANOVA with Bonferroni’s multiple comparison post-test, ns, not significant, *p < 0.05, **p < 0.01, ***p < 0.001

Using immunofluorescence staining, we found that silencing of MNX1-AS1 inhibited PKM2 and importin α5 colocalization (Fig. 5A and S5B). Moreover, immunoprecipitation assays evaluating interactions between PKM2 and importin α5 showed that MNX1-AS1 depletion significantly attenuated their respective associations (Fig. 5B, C, S5C and S5D). As a complementary approach, we used the mammalian two-hybrid system which evaluates protein-protein interactions in vivo. Bait (BIND) and prey (ACT) fusion vectors were constructed to test interactions between PKM2 and importin α5. Consistently, MNX1-AS1 silencing reduced the luciferase reporter activity produced from interactions between PKM2 and importin α5 (Fig. 5D). Collectively, these data support the notion that MNX1-AS1 acts as a scaffold to facilitate interactions between PKM2 and importin α5.

After establishing links between MNX1-AS1, PKM2 and importin α5, we turned our efforts towards identifying whether the enhancement effect of MNX1-AS1 on aerobic glycolysis was mediated by PKM2 or not. We then checked the ECAR in Huh7 cells after MNX1-AS1 overexpression combined with PKM2 knockdown. Notably, depletion of PKM2 abolished the increased level of ECAR after ectopic expression of MNX1-AS1 (Fig. 5E, F and S5E). Similarly, when PKM2 was silenced, ectopic expression of MNX1-AS1 failed to promote increases in extracellular lactate and glucose uptake levels (Fig. 5G and H). Collectively, these data establish that MNX1-AS1 implicated in the aerobic glycolysis dependent on PKM2.

Since EGFR activation facilitates the nuclear translocation of PKM2, we therefore sought to demonstrate the role of MNX1-AS1 in this process. Consistent with previous studies, EGF treatment of HepG2 cells promoted the nuclear translocation PKM2, but this process was markedly attenuated after depletion of MNX1-AS1 (Fig. 5I and S5F). Additional immunofluorescence staining analysis showed similar results where MNX1-AS1 silencing significantly decreased nucleus PKM2 signals under basal and EGFR activation conditions (Fig. 5J and K). Moreover, overexpression of MNX1-AS1 served to elevate the nuclear expression levels of PKM2 (Fig. S5G). Taken together, these data support the conclusion that MNX1-AS1 enhances PKM2 nuclear translocation in response to EGF.

### An EGF-c-Myc-MNX1-AS1 axis promotes the Warburg effect through non-glycolytic functions of PKM2

**Fig. 6:**
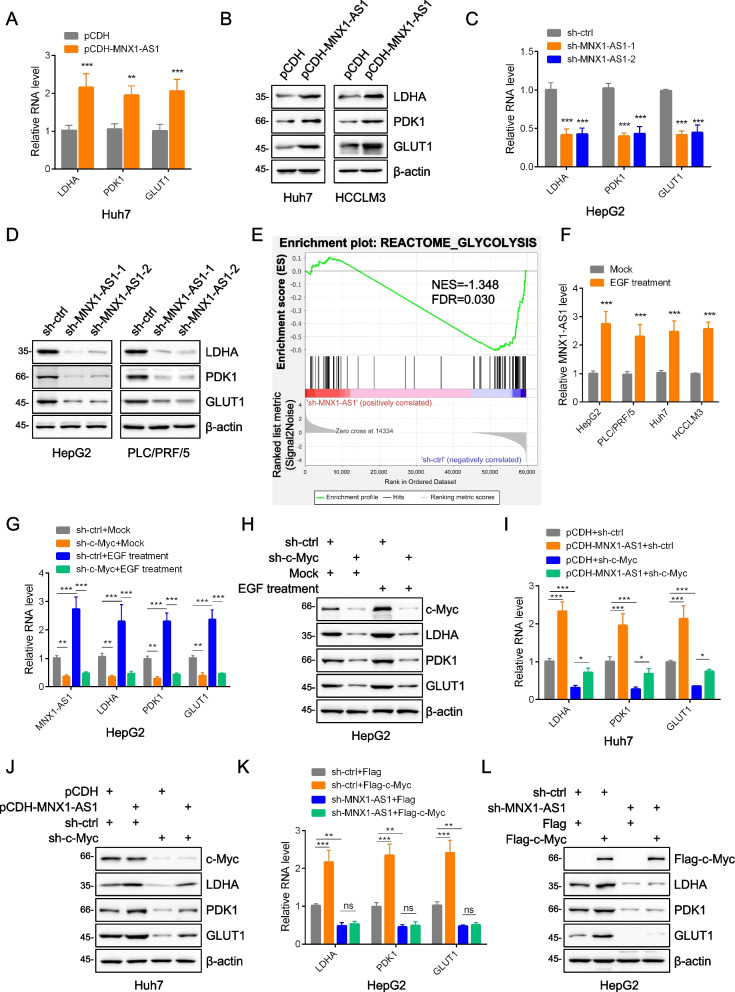
An EGF-c-Myc-MNX1-AS1 axis promotes the Warburg effect through non-glycolytic functions of PKM2. **a**-**d** Huh7 and HCCLM3 cells were infected with pCDH or pCDH-MNX1-AS1 lentiviruses, and HepG2 and PLC/PRF/5 cells were infected with sh-ctrl or sh-MNX1-AS1 lentiviruses. Huh7 and HepG2 cells were subjected to qPCR to determine the expression of LDHA, PDK1 and GLUT1 (**A** and **C**). Huh7, HCCLM3, HepG2 and PLC/PRF/5 were subsequently subjected to Western blotting to determine the expression of indicated proteins (**B** and **D**). **e** GSEA analysis of the GLYCOLYSIS pathway in sh-ctrl versus sh-MNX1-AS1 infected HepG2 cells. **f** MNX1-AS1 levels were measured by qPCR in HepG2, PLC/PRF/5, Huh7 and HCCLM3 cells after treatment with EGF (100 ng/ml) for 24 hours. **g** and **h **HepG2 cells infected with sh-ctrl or sh-c-Myc lentiviruses were subjected to mock or EGF (100 ng/ml) treatment for 24 hours before qPCR analysis of RNA level of MNX1-AS1, LDHA, PDK1 and GLUT1 (**G**) and Western blotting to determine the expression of indicated proteins (**H**). **i** and **j** Huh7 cells infected with sh-ctrl or sh-c-Myc lentiviruses were subjected to infection of pCDH control or pCDH-MNX1-AS1 lentiviruses. qPCR analysis of RNA level of LDHA, PDK1 and GLUT1 (**I**) and Western blotting to determine the expression of indicated proteins (**J**). **k** and **l** HepG2 cells infected with sh-ctrl or sh-MNX1-AS1 lentiviruses were subjected to transfection of Flag control or Flag-c-Myc. qPCR analysis of RNA level of LDHA, PDK1 and GLUT1 (**K**) and Western blotting to determine the expression of indicated proteins **L**. **A**, **C**, **F**, **G**, **I** and **K** are mean ± SD; *n* = 3 independent experiments, two-way ANOVA with Bonferroni’s multiple comparison post-test, ns, not significant, *p < 0.05, **p < 0.01, ***p < 0.001. **B**, **D**, **H**, **J** and **L** are represent of three independent experiments

After establishing the link between MNX1-AS1 and the nuclear translocation of PKM2, we turned our efforts towards identifying the downstream consequences. Considering our initial observation that MNX1-AS1 influenced glycolytic flux, we assessed how MNX1-AS1 expression influenced the expression of LDHA, PDK1 and GLUT1, three key genes known to be transcriptionally regulated by nuclear-localized PKM2 [11]. Remarkably, MNX1-AS1 overexpression increased the mRNA and protein levels of LDHA, PDK1 and GLUT1 in Huh7 and HCCLM3 cells (Fig. 6A, B and S6A) while MNX1-AS1 depletion in HepG2 and PLC/PRF/5 cells decreased their expression (Fig. 6C, D and S6B). To gain a broader picture of the effects of MNX1-AS1, we also performed RNA-seq analysis. Notably, following MNX1-AS1 knockdown, gene set enrichment analysis (GSEA) showed significant enrichment of the REACTOME_GLYCOLYSIS gene signature (Fig. 6E). Together, these data demonstrate that MNX1-AS1 promotes glycolysis by positively influencing the expression of many key pathway genes.

Since we had shown that c-Myc was responsible for the transactivation of MNX1-AS1, we sought to clarify the relationship between c-Myc, MNX1-AS1 and glycolysis. We first checked whether c-Myc affected the expression of LDHA, PDK1 and GLUT1. As expected, depletion of c-Myc phenocopied the effects of MNX1-AS1 knockdown, decreasing the mRNA levels of LDHA, PDK1 and GLUT1 in HepG2 and PLC/PRF/5 cells (Fig. S6C and S6D). In contrast, elevating c-Myc expression increased their levels (Fig. S6E and S6F). And since the function of MNX1-AS1 was integrally linked to EGFR signaling, we sought to confirm if EGF promoted c-Myc and MNX1-AS1 expression. Indeed, we found that EGF treatment significantly increased MNX1-AS1 and c-Myc mRNA levels in four different HCC cell lines (Fig. 6F and S6G). Thus, EGFR pathway activation drives c-Myc and MNX1-AS1 expression.

Lastly, we sought to amalgamate all key findings revealed by our study, namely the contribution of EGF signaling to the expression of c-Myc and MNX1-AS1, the effects of MNX1-AS1 on PKM2 nuclear translocation and the links to the Warburg effect. Towards this, we assessed the effects of EGF stimulation in c-Myc-depleted HepG2 cells. Instructively, we observed that depletion of c-Myc abolished the increased levels of MNX1-AS1 and LDHA, PDK1 and GLUT1 mRNAs resulting from EGF treatment (Fig. 6G). Accordingly, protein level measurements by Western blotting demonstrated similar changes (Fig. 6H). However, the diminished levels of LDHA, PDK1 and GLUT1 in c-Myc depleted cells could be partially rescued by overexpression of MNX1-AS1 (Fig. 6I and J). Conversely, when MNX1-AS1 was silenced, ectopic expression of c-Myc failed to promote increases in LDHA, PDK1 and GLUT1 expression (Fig. 6K and L). Moreover, akin to previous results, knockdown MNX1-AS1 and/or c-Myc significantly attenuated nuclear translocation of PKM2 induced by EGF treatment (Fig. S6H and S6I). Furthermore, we also observed that depletion of PKM2 abolished increased mRNA and protein levels of LDHA, PDK1 and GLUT1 after ectopic expression of MNX1-AS1 (Fig. S6J and S6K). Similarly, knockdown of PKM2 also attenuated increased mRNA and protein levels of LDHA, PDK1 and GLUT1 induced by EGF treatment (Fig. S6L and S6M). Thus, while c-Myc is clearly important in driving MNX1-AS1 expression, the downstream effects are not dependent on c-Myc expression per se. Consequently, these data reveal the existence of an EGF-c-Myc-MNX1-AS1 axis which promotes the expression of genes that enhance glycolytic flux through non-glycolytic functions of PKM2, thereby promoting the Warburg effect.

### Biological implications of MNX1-AS1 in tumorigenesis

**Fig. 7:**
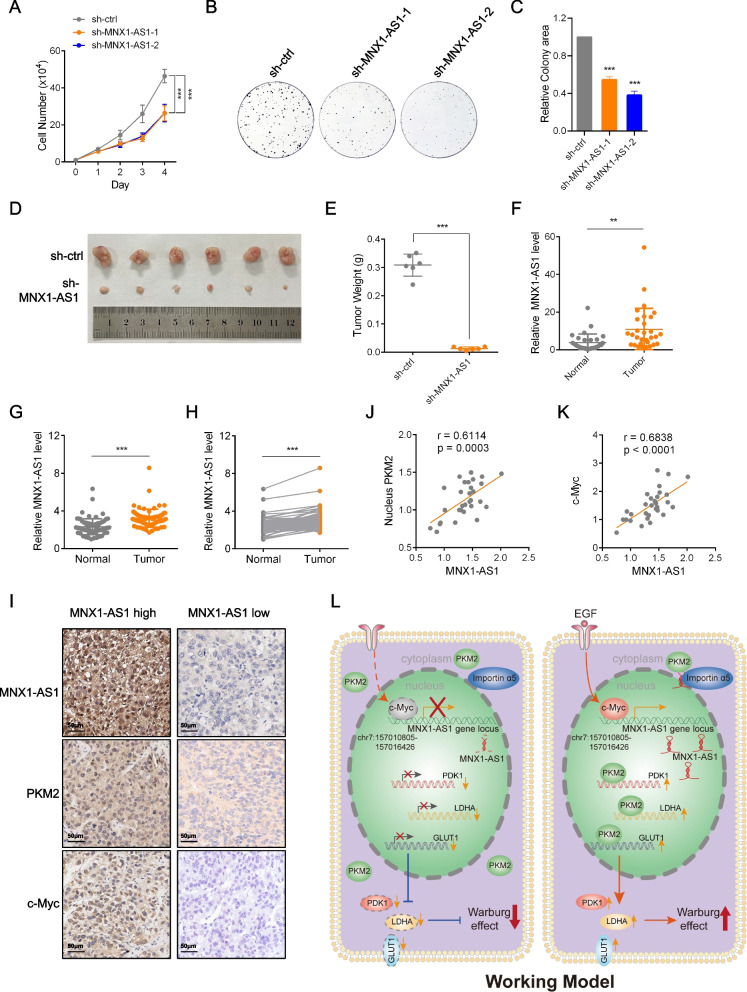
Biological implications of MNX1-AS1 in tumorigenesis. **a**-**c** Cell proliferation rates assessed by cell number determination (**A**) or clonogenic assays (**B** and **C**) in HepG2 cells stably expressing sh-ctrl or sh-MNX1-AS1. Data are shown as mean ± SD; *n* = 3 independent experiments, two-way ANOVA with Bonferroni’s multiple comparison post-test (**A**), one-way ANOVA with Tukey’s multiple comparison post-test (**C**), ***p < 0.001. **d** and **e **Comparison of the growth of PLC/PRF/5 xenografts stably expressing sh-ctrl or sh-MNX1-AS1 vectors (**D**). Final tumor weights after dissection were compared (**E**). Data are shown as mean ± SD; *n* = 6 mice per group, two-tailed Student’s t-test, ***p < 0.001. **f** qPCR assays to determine MNX1-AS1 expression in 30 pairs of hepatocellular carcinoma tumor samples and adjacent normal tissues. Data are shown as mean ± SD; *n* = 30, two-tailed Student’s t-test, **p < 0.01. **g** and **h** In situ hybridization showing higher MNX1-AS1 expression in tumors compared to adjacent normal tissues (*n* = 75), quantification of IHC staining in tumors and normal tissues (**G**), and pairwise comparisons of MNX1-AS1 expression (**H**). Data are shown as mean ± SD; *n* = 75, two-tailed paired Student’s t test, ***p < 0.001. **i**-**k** In situ hybridization of MNX1-AS1 expression and immunostaining of PKM2 and c-Myc in hepatocellular carcinoma tumor samples, (**I**) showing the representative in situ hybridization staining. Correlation analyses conducted between MNX1-AS1 and nucleus PKM2 levels (J), MNX1-AS1 and c-Myc levels (**K**), (*n* = 30). r, Pearson correlation coefficients (r) and p, *p*-values. (**l**) **l** Schematic illustration showing the working model for MNX1-AS1 in regulation of Warburg effect by promoting PKM2 nuclear translocation

We returned to consider physiological contribution of MNX1-AS1 in tumorigenesis. Unsurprisingly, depletion of MNX1-AS1 inhibited HepG2 cell proliferation while its enforced expression promoted proliferation (Fig. 7A and S7A). Moreover, these effects were mirrored in the long-term survival of HepG2 cells in clonogenic assays (Fig. 7B, C, S7B and S7C). Similarly, in the context of tumor xenografts, manipulating MNX1-AS1 expression by knockdown or overexpression indicated that MNX1-AS1 was positively correlated with the rates of in vivo tumor growth (Fig. 7D, E, S7D and S7E). Furthermore, we found that depletion of PKM2 abolished increased levels of cell proliferation and clonogenicity triggered by ectopic expression of MNX1-AS1 (Fig. S7F-H). Thus, MNX1-AS1 functions as a driver of tumor cell growth both in vitro and in vivo.

We then sought to establish whether MNX1-AS1 potentially contributed to tumorigenesis in clinical cases of hepatocellular carcinoma. Towards this, we undertook qPCR-based analyses comparing the expression of MNX1-AS1 in hepatocellular cancer tissues versus paired adjacent non-cancerous tissues. Among 30 pairs of tissues, we found an overall significant increase in MNX1-AS1 expression in hepatocellular carcinoma tissues (Fig. 7F). Using an independent cohort of 75 pairs of hepatocellular carcinoma tumors and adjacent non-cancerous tissues, we employed in situ hybridization (ISH) to measure MNX1-AS1. As per the qPCR data, the levels of MNX1-AS1 were increased in tumor tissues where a clearly nuclear pattern of staining was evident (Fig. 7G and S7I). Moreover, pairwise comparisons showing MNX1-AS1 was increased in 72/75 (96%) of tumors relative to normal tissues (Fig. 7H). Finally, in order to establish evidence from clinical samples showing that MNX1-AS1 expression in hepatocellular carcinoma was related to nucleus PKM2 and c-Myc levels. Towards this, we employed in situ hybridization (ISH) and immunohistochemistry (IHC) to measure MNX1-AS1 expression, PKM2 and c-Myc levels. Consistent with the mechanism disclosed in the in vitro studies, ex vivo tissues with high MNX1-AS1 levels showed high nucleus PKM2 and c-Myc expression (Fig. 7I), providing an overall positive correlation between nucleus PKM2 or c-Myc levels and MNX1-AS1 expression (Fig. 7J and K).

Taken together, our data discloses a role for MNX1-AS1 as an oncogene, functioning to promote the Warburg effect in HCC cells. Linked to EGF signaling and transactivated by c-Myc, MNX1-AS1 enables the nuclear import of PKM2, unleashing its non-glycolytic function as a coactivator to activate transcription of *LDHA*, *PDK1* and *GLUT1* genes. Elevated expression of LDHA, PDK1 and GLUT1 was known to promote the Warburg effect [11, 30]. Our working model is illustrated in Fig. 7L.

## Discussion

MNX1-AS1 has previously attracted the attention of researchers with different studies showing it is overexpressed in a variety of malignancies including colorectal cancer, cervical cancer, ovarian cancer, prostate cancer, breast cancer, esophageal cancer (ESCC), laryngeal squamous cell carcinoma (LSCC), lung cancer, gall bladder cancer (GBC), hepatocellular carcinoma and intrahepatic cholangiocarcinoma [19–26, 31–34]. Within these reports there is evidence that higher MNX1-AS1 expression is correlated with detrimental pathological features and worse clinical outcomes. Here our findings corroborated the overexpression of MNX1-AS1 in HCC, and we further established that MNX1-AS1 is differentially overexpressed in greater than 20 cancer types. These findings position MNX1-AS1 as a true pan cancer-expressed lncRNA, highlighting the importance of understanding how it contributes to tumorigenesis. As with proteins, lncRNAs are multifunctional entities and it therefore not surprising that several mechanisms of action have been attributed to MNX1-AS1.

Presently, the majority of published studies ascribe MNX1-AS1 to be a competing endogenous RNA (ceRNA), acting against different targets to elicit downstream effects on the migration and invasion of HCC [19], ESCC [31], ovarian [22] and lung cancers [32] by sponging miR-218-5p, miR-34a, miR-744-5p and miR-527, respectively, while in LSCC, MNX1-AS1 was shown to promote β-catenin signaling by targeting miR-744–5p [34]. Other reports propose that MNX1-AS1 can directly or indirectly influence major signaling pathways including Jak/Stat3 and Akt/mTOR in breast cancer [22, 35], MAPK in cervical cancer [24] and also possibly Notch in LSCC [36]. These functions are presumably carried out in the cytoplasm while we found there was a striking presence of MNX1-AS1 in the nucleus of ex vivo HCC tissues. We also observed a predominantly cytoplasmic pool of MNX1-AS1 in HCC cell lines under basal conditions, but its nuclear localization dramatically increased after EGF treatment. Only two current reports definitively indicate that MNX1-AS1 functions in the nucleus. Wu et al. showed that MNX1-AS1 functioned to activate the co-transcriptional/translation factor Y-box-binding protein 1 (YB1), with MNX1-AS1 transactivated by c-Myc but with YB1 involved in promoting downstream c-Myc target expression [26]. Alternatively, Li et al. showed that MNX1-AS1 was coopted to occupy the MNX1 gene promoter along with c-Myc and MAZ to increase MNX1 expression. In turn, MNX1 serves to drive expression of the Hippo inhibitor Ajuba, thereby triggering downstream Hippo target expression. Our report now establishes a very different role for MNX1-AS1 in the nucleus through PKM2, also invoking a role for c-Myc but with important differences from previous reports.

As the major PK isoform expressed by cancer cells, PKM2 is a key enzyme involved in promoting tumorigenesis through its contributions to glycolysis and the Warburg effect [37]. However, not only is PKM2 an implicit component of the glycolytic cascade but it has non-glycolytic functions. It is well known that c-Myc regulates PKM2 transcription, with nucleus PKM2 creating a positive feedback-loop to induce c-Myc expression [12, 38]. Furthermore, PKM2 is also embedded within HIF-1α signaling with HIF-1α transcriptionally regulates PKM2 expression, with the nuclear-localized pool of PKM2 functioning as a HIF-1α coactivator, influencing the transcription of key genes associated with glycolysis including *LDHA*, *PDK1* and *GLUT1* [11]. We found that EGFR activation increased MNX1-AS1 expression through c-Myc, and notably, EGFR activation is known to promote PKM2 nuclear translocation to enhance the Warburg effect [12]. While our data agree that MNX1-AS1 expression is induced by c-Myc, a major exception from previous reports was that the downstream effects of MNX1-AS1 were independent of c-Myc. Furthermore, MNX1-AS1 does not influence PKM2 protein levels, strengthening the notion that its primary role is to facilitate the PKM2 nuclear translocation.

Prior work has shown that interactions between PKM2, importin α5 and PIN1 are essential for PKM2 nuclear translocation [12]. Here we established that MNX1-AS1 directly bridges interactions between PKM2 and importin α5, with sequences embedded in exons 1 and 2 of MNX1-AS1 responsible for binding to importin α5 and PKM2, respectively. On the other hand, the MNX1-AS1 binding sequences within PKM2 and importin α5 overlapped those previously established as containing the NLS of each protein. And in response to EGFR activation, the scaffolding function of MNX1-AS1 was responsible for the entry of PKM2 into the nucleus. We did not find any obvious role for PIN1 in this process, but it remains conceivable that our results reflect a similar or even the same mechanism. Notwithstanding this point, we showed remarkably that manipulating MNX1-AS1 in HCC cells produced glycolytic gene changes consistent with the reported effects of nucleus PKM2.

Certain lncRNAs have already been shown to engage PKM2 and affect its activity in cancer cells in remarkably different manners. The examples of the lncRNAs FEZF1-AS1 and HULC already described [16, 17] revealed direct binding as adapters consequently promoting PKM2 expression and/or activity to increase glycolytic flux. Of note, exon 10 in PKM2 was responsible for HULC binding with the same sequences included in the PKM2 region we found to bind to MNX1-AS1. Alternatively, lncRNAs can cause PKM2 to “switch sides”, changing substrate preference from PEP to proteins such as reported with lncCCAT1. Namely, the interaction of lncCCAT1 with PKM2 promotes it to alternatively phosphorylate and activate SREBP2, leading to transcriptional enhancement of lipogenic genes including those promoting the Warburg effect [39]. This report now expands this repertoire to illustrate in detail the mechanism whereby MNX1-AS1 promotes the nuclear functions of PKM2.

Lastly, it is pertinent to consider the utility of our findings. Given the accumulating evidence, it seems indisputable that MNX1-AS1 is important for the tumorigenesis of many cancer types, not just HCC as studied here. This proposes that the elevated expression of MNX1-AS1 could broadly serve as a prognostic biomarker or even provide therapeutic opportunities as a drug target. The latter is interesting to ponder given the difficulties associated with targeting glycolysis which is a universal process in malignant and normal cells alike. Given aberrant MNX1-AS1 expression as a common event in cancer cells, this suggests potential therapeutic opportunities to counter glycolysis in cancer cells via targeting the non-glycolytic functions of key enzymes such as PKM2. And although glycolytic enzymes are highly abundant in cells, with most of PKM2 found cytosolically, the contribution of the nuclear pool towards reinforcing the aerobic glycolytic program in cancer cells appears to be of considerable significance. Indeed, we found that targeting MNX1-AS1 abolished EGF-stimulated PKM2 nuclear translocation with robust inhibition of tumor growth observed in vitro and in vivo. It can also be noted there is considerable interest in the therapeutic targeting of EGFR signaling which is activated in multiple cancers [40, 41]. Our discovery of the role of elevated MNX1-AS1 therefore provides opportunities to counter EGFR activation.

## Conclusions

In conclusion, our works demonstrated that MNX1-AS1 as a pan-cancer upregulated lncRNA, reinforces the Warburg effect through facilitating the non-glycolytic actions of PKM2 in the cell nucleus in response to EGF. Thus implicitly highlights the potential of targeting MNX1-AS1 to selectively counter the Warburg effect in a range of tumor types.

## Supplementary Information

**Additional file 1.**

**Additional file 2.**

**Additional file 3.**

**Additional file 4.**

**Additional file 5.**

## Data Availability

All data generated or analyzed during this study are included in this published article and its Additional Files. The RNA-seq data (sh-ctrl versus sh-MNX1-AS1) was submitted to GEO (Gene Expression Omnibus) database (Accession number: GSE213064).

## Abbreviations

LncRNA: long non-coding RNA
HCC: hepatocellular carcinoma
MNX1-AS1: motor neuron and pancreas homeobox 1 antisense RNA 1
EGFR: epidermal growth factor receptor
PKM2: pyruvate kinase isoform M2
qPCR: quantitative reverse transcription polymerase chain reaction
FISH: fluorescence in situ hybridization
RIP: RNA immunoprecipitation
ChIP: chromatin immunoprecipitation
MS: mass spectrometry
ECAR: extracellular acidification rate
GSEA: gene set enrichment analysis
IF: immunofluorescence
PIN1: peptidylprolyl cis/trans isomerase, NIMA-interacting 1
