# Supplementary material for: MNX1-AS1, a c-Myc induced lncRNA, promotes the Warburg effect by regulating PKM2 nuclear translocation
Source: J Exp Clin Cancer Res. 2022 Dec 7;41:337. doi: 10.1186/s13046-022-02547-3 (PMC9727912; doi:10.1186/s13046-022-02547-3)
Supplement: Supplementary file 4 — Additional file 4. [file 13046_2022_2547_MOESM4_ESM.pdf]

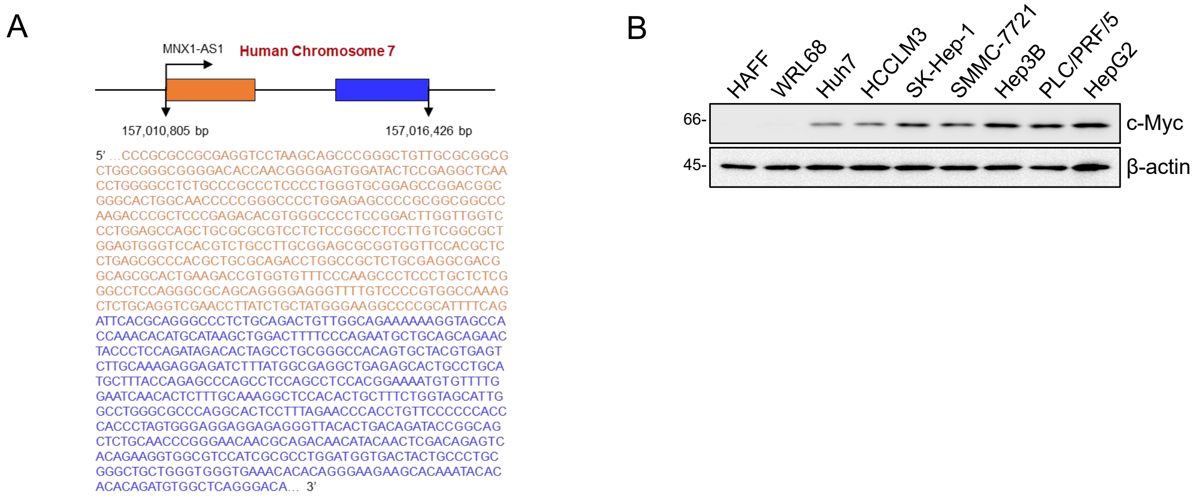

Fig. S1 MNX1-AS1 is a c-Myc associated pan-cancer lncRNA

A. Schematic illustration of the intron-exon structure of MNX1-AS1 on chromosome 7.

B. Western blot analysis measuring c-Myc protein levels in hepatocellular carcinoma and normal cell lines. Data shown represent three independent experiments.

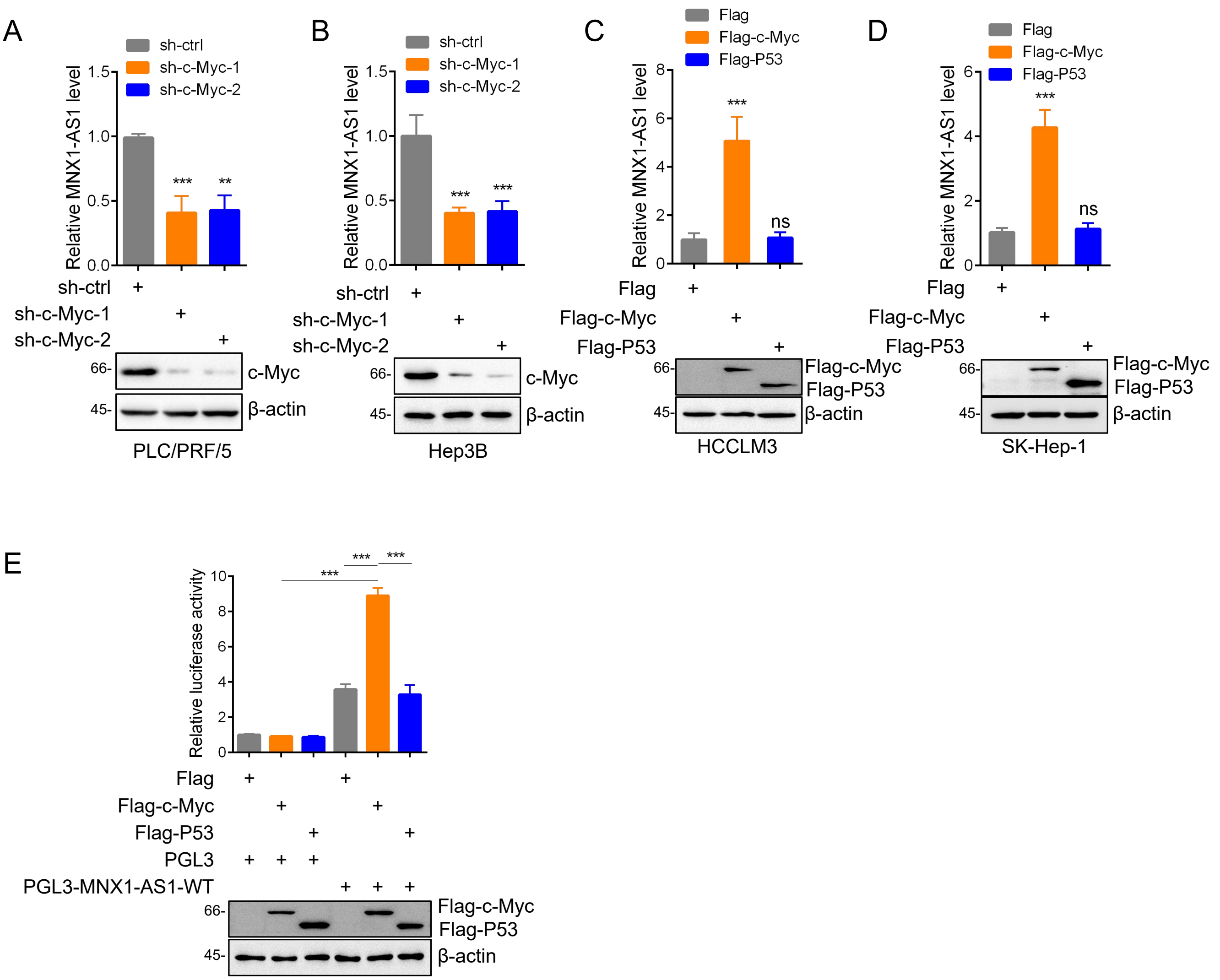

Fig. S2 MNX1-AS1 is a direct target of c-Myc

A-D. MNX1-AS1 levels were measured by qPCR after infection of PLC/PRF/5 cells or Hep3B cells with lentiviruses containing control shRNA (sh-ctrl) or two independent shRNAs targeting c-Myc (A and B), or after transfection of HCCLM3 cells or SK-Hep-1 with either control Flag plasmid, Flag-c-Myc or Flag-P53 (negative control) plasmids (C and D).

E. Luciferase reporter assays in HepG2 cells after co-transfection of indicated plasmids for 24 h. The expression of Flag-c-Myc and Flag-P53 were confirmed by Western blotting.

A-E. are mean ± SD; n=3 independent experiments, one-way ANOVA with Tukey's multiple comparison post-test, ns, not significant, ***p < 0.001.

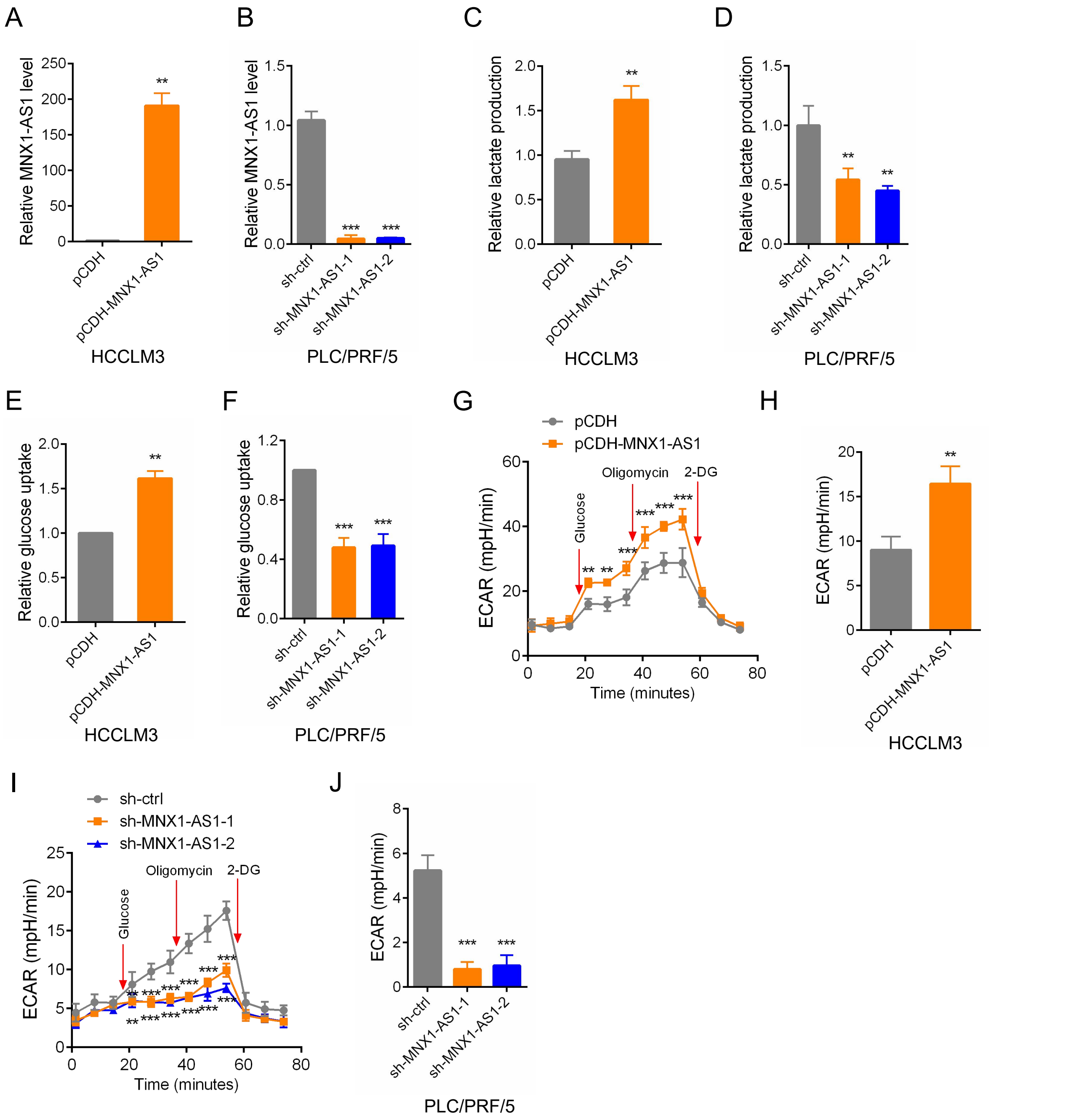

Fig. S3 MNX1-AS1 promotes glycolysis in HCC

A and B. MNX1-AS1 levels were measured by qPCR after infection of HCCLM3 cells with either pCDH control or pCDH-MNX1-AS1 lentiviruses (A), or after infection of PLC/PRF/5 cells with lentiviruses containing control shRNA (sh-ctrl) or two independent shRNAs targeting MNX1-AS1 (B).

C and D. Extracellular lactate production in HCCLM3 (C) and PLC/PRF/5 (D) cells from (A) and (B).

E and F. Glucose uptake were measured in HCCLM3 (E) and PLC/PRF/5 (F) cells from (A) and (B).

G – J. ECAR was measured by Seahorse XF assays in HCCLM3 (G and H) and PLC/PRF/5 (I and J) cells from (A) and (B). The glycolysis rate were calculated as: (Maximum ECAR before Oligomycin injection)-(Last ECAR before Glucose injection).

A, C, E and H. are mean ± SD; n=3 independent experiments, two-tailed paired Student's t test, $**p < 0.01$. (B, D, F and J) are mean ± SD; n=3 independent experiments, one-way ANOVA with Tukey's multiple comparison post-test, $**p < 0.01$, $***p < 0.001$. (G and I) are mean ± SD; n=3 independent experiments, two-way ANOVA with Bonferroni's multiple comparison post-test, ns, not significant, $**p < 0.01$, $***p < 0.001$.

A

Protein	prot_ cover	Unique Peptides	MW [kDa]
PKM2	61	27	57.9
KPNA1(importin α5)	52	23	60.2
ENO1	50	12	47.1

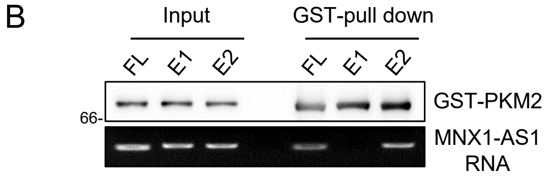

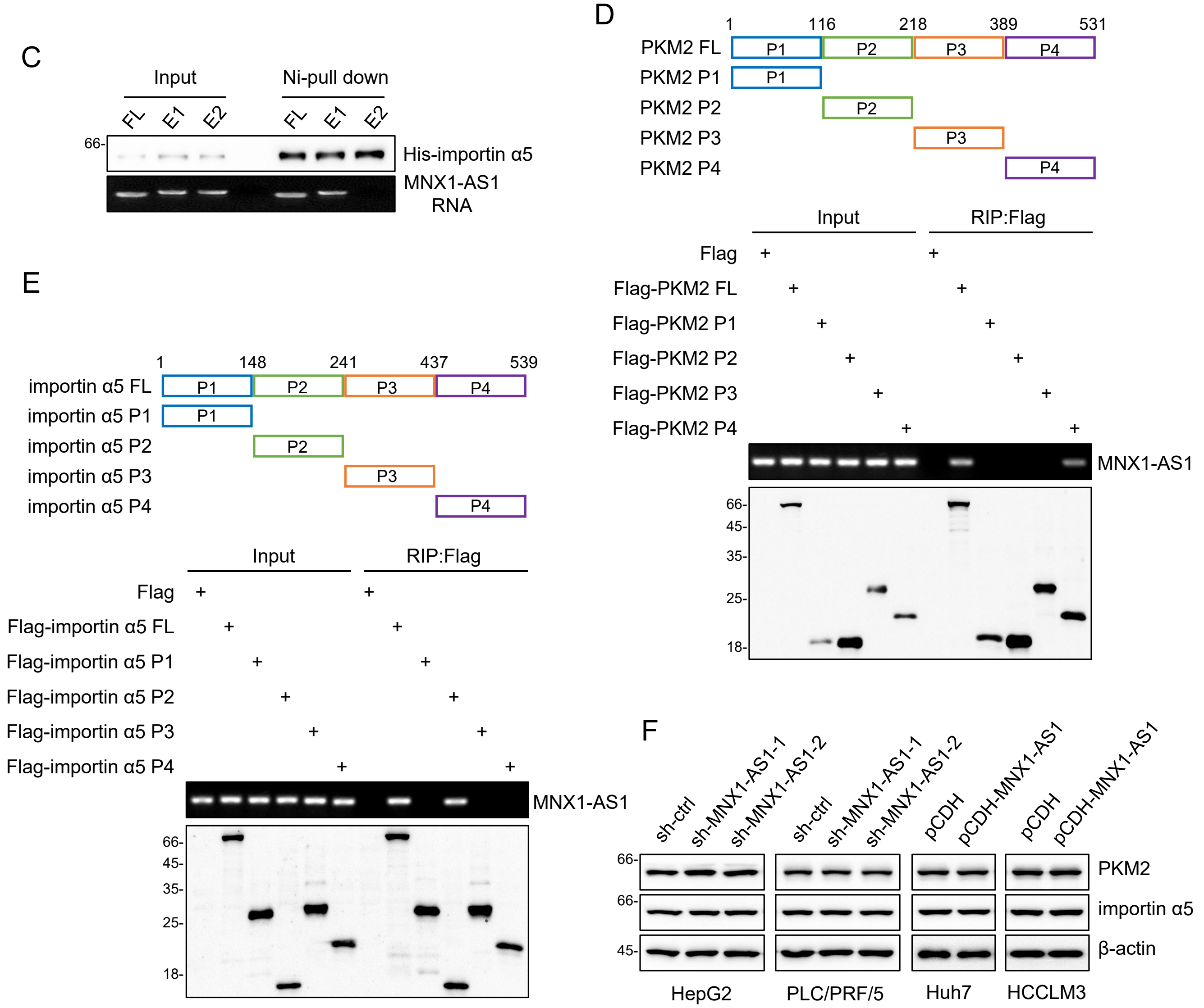

Fig. S4 MNX1-AS1 directly binds to PKM2 and importin α5

A . Table showing selected MNX1-AS1 interacting proteins involved in glycolysis identified by mass spectrometry analysis.

B and C. RNA protein pull-down assays were conducted with glutathione Sepharose (B) and Ni-NTA Sepharose (C), results were assessed by semi-quantitative RT-PCR using primers detecting Full-length, Exon 1 or Exon 2 of MNX1-AS1.

D and E. Schematic illustration of PKM2 and importin α5 functional domains and corresponding truncation constructs (top D, E). RIP assays were conducted using Flag antibody with cell lysates from HepG2 cells transfected with full-length PKM2 or its respective deletion mutants (D), importin α5 or its respective deletion mutants (E). Co-precipitated proteins were revealed by Western blot using anti-Flag-HRP antibody. MNX1-AS1 was assessed by semi-quantitative RT-PCR.

F. HepG2 and PLC/PRF/5 cells bearing either sh-ctrl or sh-MNX1-AS1 lentiviruses (left), or Huh7 and HCCLM3 cells infected with either pCDH or the pCDH-MNX1-AS1 overexpression vector (right). PKM2 and importin α5 were assessed by Western blot analysis.

B-F. represent three independent experiments.

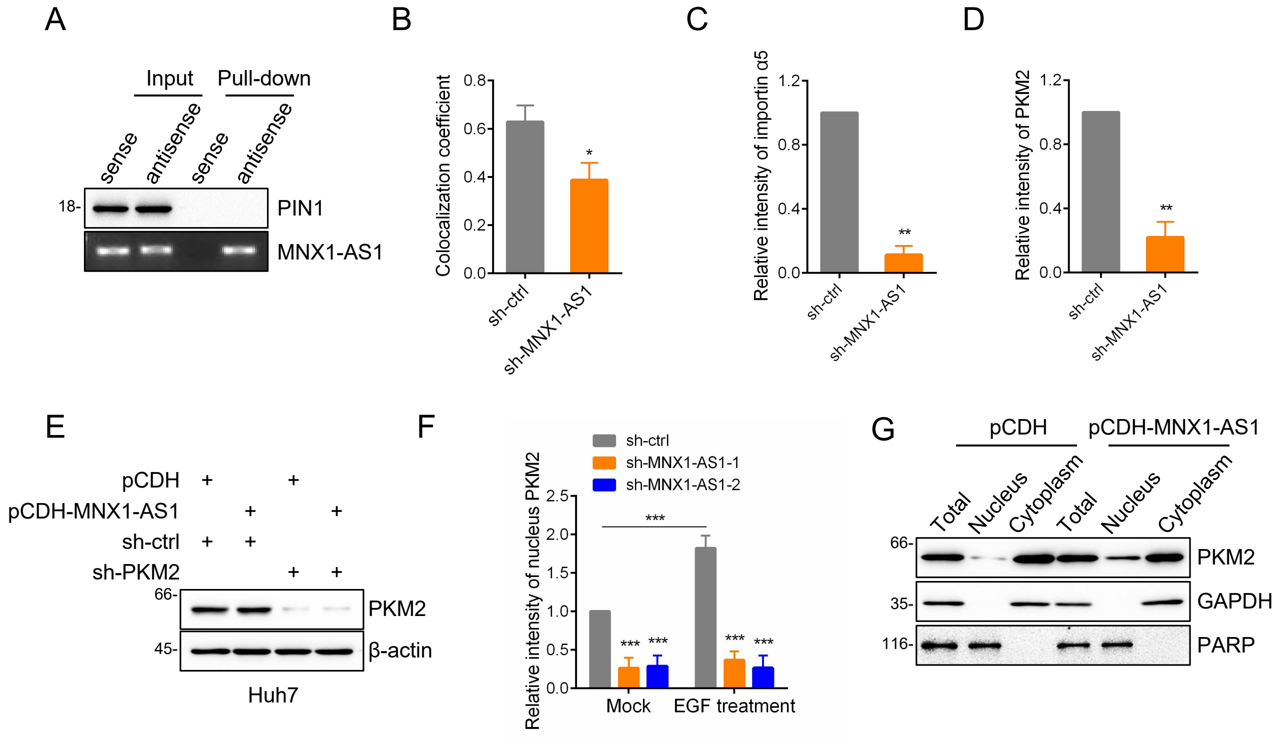

Fig. S5 MNX1-AS1 enhances PKM2 nuclear translocation in response to EGF

A . RNA protein pull-down assays using biotin-labelled sense or anti-sense MNX1-AS1 probes against cell lysates from HepG2 cells, RNA protein pull-down assay samples were subjected to Western blotting against PIN1 (top) or semi-quantitative RT-PCR for MNX1-AS1 (bottom).

B. Colocalization between PKM2 and importin α5 in Figure 5 was measured using ZEN imaging software.

C-D. Quantitation of the experiments in Figures 5B (C) and 5C (D) using densitometric measurements using ImageJ software.

E. Western blot analysis to verify PKM2 knockdown.

F. Quantitation of the experiments in Figures 5I using densitometric measurements using ImageJ software.

G. Huh7 cells were infected with lentiviruses containing pCDH control or pCDH-MNX1-AS1. Cells were subjected to subcellular fractions, and PKM2 subcellular location were assessed by Western blot analysis. PARP and GAPDH served as controls for Western blotting.

(A, E and G) represent three independent experiments. (B-D) are mean ± SD; n=3 independent experiments, two-tailed paired Student's t test, *p < 0.05, **p < 0.01. (F) is mean ± SD; n=3 independent experiments, two-way ANOVA with Bonferroni's multiple comparison post-test, ***p < 0.001.

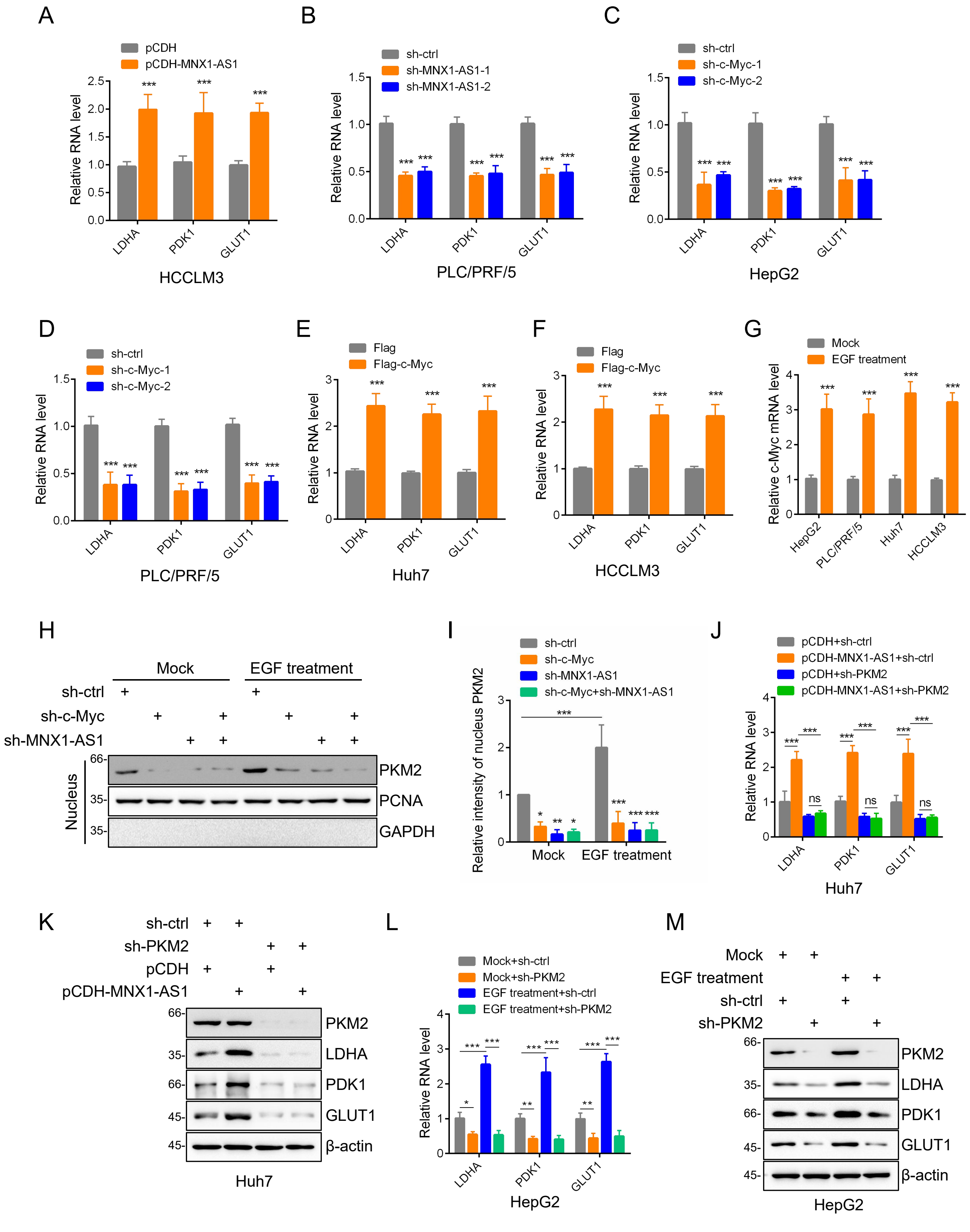

(legend on next page)

Fig. S6 An EGF-c-Myc-MNX1-AS1 axis promotes the Warburg effect through non-glycolytic functions of PKM2

A . HCCLM3 cells infected with pCDH control or pCDH-MNX1-AS1 lentiviruses and subsequently subjected to qPCR to determine the expression of LDHA, PDK1 and GLUT1.

B. PLC/PRF/5 cells were infected with lentiviruses containing sh-ctrl or sh-MNX1-AS1. Cells were subsequently subjected to qPCR to determine the expression of LDHA, PDK1 and GLUT1.

C-F. HepG2 and PLC/PRF/5 cells were infected with lentiviruses containing sh-ctrl or sh-c-Myc (C and D), or Huh7 and HCCLM3 cells were transfected with Flag control or Flag-c-Myc (E and F). Cells were subsequently subjected to qPCR to determine the expression of LDHA, PDK1 and GLUT1.

G. c-Myc mRNA levels were measured by qPCR in HepG2, PLC/PRF/5, Huh7 and HCCLM3 cells after treatment with EGF (100 ng/ml) for 24 hours.

H-I. HepG2 cells were infected with lentiviruses containing sh-ctrl, sh-c-Myc and/or sh-MNX1-AS1. Cells were with and without EGF (100 ng/ml) treatment before subcellular fractions. Nucleus PKM2 were assessed by Western blot analysis (H). Quantitation of the experiments using densitometric measurements (I).

J and K. Huh7 cells infected with sh-ctrl or sh-PKM2 lentiviruses were subjected to infection of pCDH control or pCDH-MNX1-AS1 lentiviruses. qPCR analysis of RNA level of LDHA, PDK1 and GLUT1 (J) and Western blotting to determine the expression of indicated proteins (K).

L and M. HepG2 cells infected with sh-ctrl or sh-PKM2 lentiviruses were subjected to mock or EGF (100 ng/ml) treatment for 24 hours before qPCR analysis of RNA level of LDHA, PDK1 and GLUT1 (L) and Western blotting to determine the expression of indicated proteins (M). (A-G, I, J and L) are mean $\pm$ SD; n=3 independent experiments, two-way ANOVA with Bonferroni's multiple comparison post-test, ns, not significant, $*p < 0.05$, $**p < 0.01$, $***p < 0.001$. (H, K and M) represent three independent experiments.

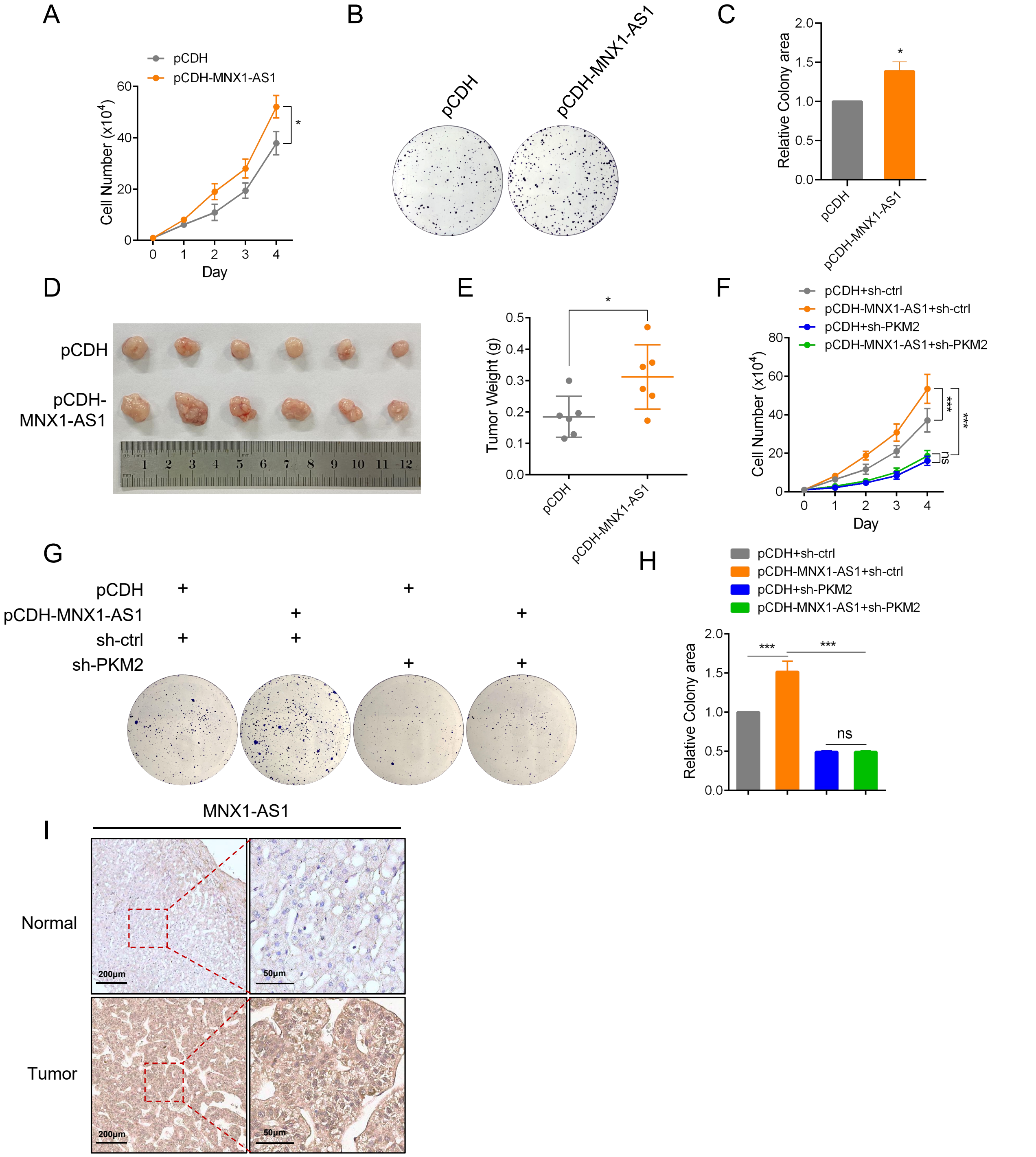

Fig. S7 Biological implications of MNX1-AS1 in tumorigenesis

A-C. Cell proliferation rates were compared by cell number determination (A) or clonogenic assays (B and C) in Huh7 cells stably expressing pCDH or pCDH-MNX1-AS1. Data are shown as mean ± SD; n=3 independent experiments, two-tailed paired Student's t test, *$p < 0.05$.

D and E. Comparison of the growth of Huh7 xenografts stably expressing pCDH or pCDH-MNX1-AS1 (D) along with final tumor weights after dissection (E). Data are shown as mean ± SD; n=6 mice per group, two-tailed Student's t-test, *$p < 0.05$.

F-H. Cell proliferation rates were compared by cell number determination (F) or clonogenic assays (G and H) in Huh7 cells infected with sh-ctrl or sh-PKM2 lentiviruses were subjected to infection of pCDH control or pCDH-MNX1-AS1 lentiviruses. (F and H) are mean ± SD; n=3 independent experiments, two-way ANOVA with Bonferroni's multiple comparison post-test, ns, not significant, ***$p < 0.001$.

I. In situ hybridization showing higher MNX1-AS1 expression in tumors compared to adjacent normal tissues (n=75), (I) showing the representative in situ hybridization staining.
